# Modeling anti-tumor immune responses using patient-derived melanoma organoids

**DOI:** 10.1007/s00262-025-04269-9

**Published:** 2025-12-23

**Authors:** Kamila Kaminska, Bengt Phung, Jacob Karlström, Martin Lauss, Katja Harbst, Teresa Svensson, Kristian Pietras, Kari Nielsen, Ana Carneiro, Henrik Ekedahl, Karolin Isaksson, Göran Jönsson

**Affiliations:** 1 Division of Oncology, Department of Clinical Science, Faculty of Medicine, Lund University, Medicon Village, 22185 Lund, Sweden; 2 Lund University Cancer Center, Lund, Sweden; 3 Department of Oncology, Skåne University Hospital, Lund, Sweden; 4 Skåne University Hospital Comprehensive Cancer Center, Lund, Sweden; 5 Division of Translational Cancer Research, Department of Laboratory Medicine, Lund University, Lund, Sweden; 6 Department of Dermatology, Helsingborg General Hospital, Helsingborg, Sweden; 7 Department of Dermatology, Skåne University Hospital, Lund, Sweden; 8 Division of Dermatology, Department of Clinical Science, Faculty of Medicine, Lund University, Lund, Sweden; 9 Department of Surgery, Skåne University Hospital, Kristianstad, Sweden; 10 Division of Surgery, Department of Clinical Science, Faculty of Medicine, Lund University, Lund, Sweden

**Keywords:** Cancer, Melanoma, Immune response, Patient-derived organoid, PD1

## Abstract

Immune checkpoint blockade (ICB) therapy can restore T cell function in tumors, but not all patients benefit, and the mechanisms behind this remain unclear. In this study, we used patient-derived organotypic (PDO) cultures from metastatic melanoma to examine transcriptomic and cellular changes following ex vivo T cell stimulation. Genomic and transcriptomic features were preserved during PDO formation, capturing melanoma heterogeneity. PDOs from ICB-responsive patients showed rapid T cell expansion upon T cell stimulation, unlike those from ICB-resistant tissue. Resistant tissue harbored T cells lacking activation and checkpoint markers, suggesting non-tumor-reactive T cells. A T cell-specific transcriptomic score, activated in responsive PDOs, correlated with improved overall and relapse-free survival in metastatic melanoma patients treated with ICB. These findings demonstrate that ex vivo analysis is a viable tool to investigate mechanisms of ICB response and may help identify predictive biomarkers for patient outcome.

**Supplementary Information:**

The online version contains supplementary material available at 10.1007/s00262-025-04269-9.

## Introduction

Melanoma, a highly aggressive form of skin cancer, poses significant challenges in treatment, especially in its metastatic stage. However, during the last decade, treatment with clinical introduction of immunomodulatory therapies has dramatically changed the outcome of melanoma patients with advanced disease [1]. PD1 blocking antibodies are currently the first-line treatment in advanced melanoma given in the adjuvant or neoadjuvant setting. Despite the central role of PD1 blockade in the clinical management of cancer patients, the functional consequences are not fully known. PD1 is expressed on activated T cells, B cells, and dendritic cells [2–4]. The ligand of PD1, PDL1, is induced on melanoma cells in response to interferon gamma (IFNG), thereby activating a negative feedback loop that inhibits tumor-specific T cells [5]. Blocking PD1 reactivates these T cells allowing tumor-specific killing to occur. However, a deeper understanding of the molecular consequences of T cell activation within tumors is still needed. Recent studies describing the superior clinical efficacy of neoadjuvant ICB compared to adjuvant ICB underscore the need to understand the early immunological changes occurring within tumors [6]. Voabil et al. [7] used patient-derived tissue fragments (PDTF) to analyze early immunological consequences of PD1 blockade. Their analysis demonstrated the expression of several chemokines such as IFNG and CXCL10, in tumor fragments from patients who responded clinically to ICB, irrespective of cancer type [7]. Alternative model systems to PDTFs include patient-derived organoids [8, 9]. These models have the advantage of preserving the tumor microenvironment allowing the evaluation of immunomodulatory effects on both tumor and immune cells. Using patient-derived organoids, Ou et al. showed that PD1 blockade activated CD8^+^ T cells and induced tumor cell death confirming the utility of such models in cancer immunology research [10]. However, the use of such ex vivo models to develop new predictive biomarkers and exploring early transcriptional effects of PD1 blockade have not been fully elucidated.

In this study, we generated patient-derived organotypic cultures from fresh metastatic melanoma tissue. These cultures preserved several immune and molecular properties ex vivo. By stimulating T cells from both ICB responders and non-responders, we identified distinct transcriptional signatures that were predictive of patient outcomes following ICB therapy. Collectively, we show that patient-derived organotypic cultures are a valuable model for studying anti-tumor immune responses and discovering new predictive biomarkers.

## Material and methods

### Patient samples and organotypic cultures

Tumor material used to establish patient-derived organotypic cultures was acquired from patients undergoing surgical resection of metastatic melanoma at Skåne University Hospital, Kristianstad General Hospital, and Helsingborg General Hospital included in BioMEL [11] (ClinicalTrials.gov ID NCT05446155). All patients undergoing metastatic melanoma surgery (years 2021–2023) from whom tissue was obtained provided written informed consent for the collection of tissue and matched normal blood samples for research as approved by the local ethical board (Lund University Ethical Review Board, Dnr. 2013/101). The study adhered to the declaration of Helsinki. Tissue biopsies were processed fresh usually within 1–2 h from the surgical procedure. Two types of patient-derived organotypic (PDO) cultures were set up from fresh tumor material: semi-solid PDO cultures modified from Vilgelm et al. [12] and air–liquid interface (ALI) cultures as in Neal et al. [13] with modifications (Supplementary information).

### T cell stimulation in patient-derived organotypic cultures

Both types of cultures were treated for 1 week with 10 µg/ml nivolumab (Selleckchem) in the presence of 2 μg/ml anti-CD3 (clone HIT3a, BioLegend) and 2 μg/ml anti-CD28 (clone CD28.2, BioLegend) antibodies. Baseline and treated samples were collected for bulk RNA sequencing.

### Genomic analyses

PDO cultures recovered from the matrix and original tumors were processed for RNA and DNA extraction with AllPrep DNA/RNA Mini Kit (Qiagen). Sequencing libraries were prepared using TruSeq Stranded mRNA Library Prep kit (Illumina), pooled and sequenced in a 2 × 150 bp paired-end setup (Illumina) on NovaSeq 6000 (Illimuna). Bulk RNA sequencing data were processed using HISAT and Stringtie [14] to obtain fragments per kilobase of transcript per million mapped reads (FPKM) values. Variants in BRAF, NRAS, and NF1 were called from hisat bam files using VarDict v.1.8.2 [15]. The resulting variants were annotated with Annovar [16]. Copy number alterations were inferred using InferCNV (version 1.18.1). An InfercnvObject was constructed from 71 bulk RNA-seq organoid samples pooled together with 15 bulk RNA-seq samples from benign nevi, which were designated as control samples. MiXCR [17] version 3.0.3 was applied to detect T cell receptor clones. VDJtools version 1.2.1 was run on MiXCR output [18] (Supplementary information).

### Immunostaining

Organoids and a fragment of original tumor tissue from each patient were fixed in 10% neutral-buffered formalin solution (Sigma) for 24 h and processed according to standard dehydration protocol and embedded in paraffin blocks. Sections were taken and stained for SOX10 (BioCare Medical, clone BC34), CD20 (Roche, clone L26), and CD8 (Cell Marque Sigma-Aldrich, clone C8/144B). Immunoslides were scanned with NanoZoomer S60 (Hamamatsu) and processed with NDP.view2.8.24 (Hamamatsu Photonics K.K.) (Supplementary information).

### Flow cytometry analysis

PDO cultures were recovered from culture matrix as described above and further dissociated into single-cell suspension. Antibodies used in cocktail in FACS buffer (1% FBS in DPBS) are: anti-CD45-APC-Vio®770 (clone 5B1; Miltenyi), anti-CD3-PE (clone BW264/56; Miltenyi), and anti-CD19-FITC (clone HIB19; BD). Samples were analyzed on FACSMelody Cell Sorter (BD Biosciences); data were analyzed with FlowJo v10.9.0.

### Single-cell RNA sequencing

We generated scRNAseq data using Chromium Next GEM Single Cell 3’ Kit (10 × Genomics) according to manufacturer’s recommendations. Libraries were sequenced on NovaSeq6000 (Illumina) as per 10 × Genomics User Guide. The h5 files were processed and merged using the R package Seurat 4.0.182; the data were reduced to protein-coding genes, translational (RPS/RPL) and mitochondrial genes (MT-), and genes which a maximum count < = 4 were removed. Cells with less than 500 expressed genes were removed. The data were normalized using SCTransform. Harmony embeddings were calculated from the principal components [19]. The data were visualized using UMAP, and clusters were identified using Seurat (Supplementary information).

### Single-cell spatial transcriptomics

Single-cell spatial transcriptomic data were obtained using the CosMx platform (Nanostring, Seattle, WA) as described previously [20]. Stromal cells were distinguished as endothelial cells and fibroblasts. Tumor cells with B2M log-transformed expression > = 3 were considered “MHC-I high”; tumor cells without any expression of MITF, TYR, MLANA, and PMEL were considered “melanin low” and else were considered “melanin high.”

### External single-cell and bulk RNA sequencing data

Single-cell RNA sequencing data from Pozniak et al. [21] were downloaded as “Entire_TME.Rds” data file. The data were normalized using SCTransform [22], and initial zero counts were restored. PCA was performed, and harmony was used to adjust for 3 ‘ vs 5 ‘ library chemistry [19]. Clustering was performed using the Seurat (Seurat 4.3.0) [23]. UMAP was used to visualize the data, and cluster identities were manually assigned using key marker gene expression. External bulk RNA sequencing data have been processed as described previously [24]. Data from Riaz et al. [25], Van Allen et al. [26], TCGA [27], and Gide et al. [28] were used (Supplementary information).

## Results

### Patient-derived melanoma organotypic cultures

**Fig. 1:**
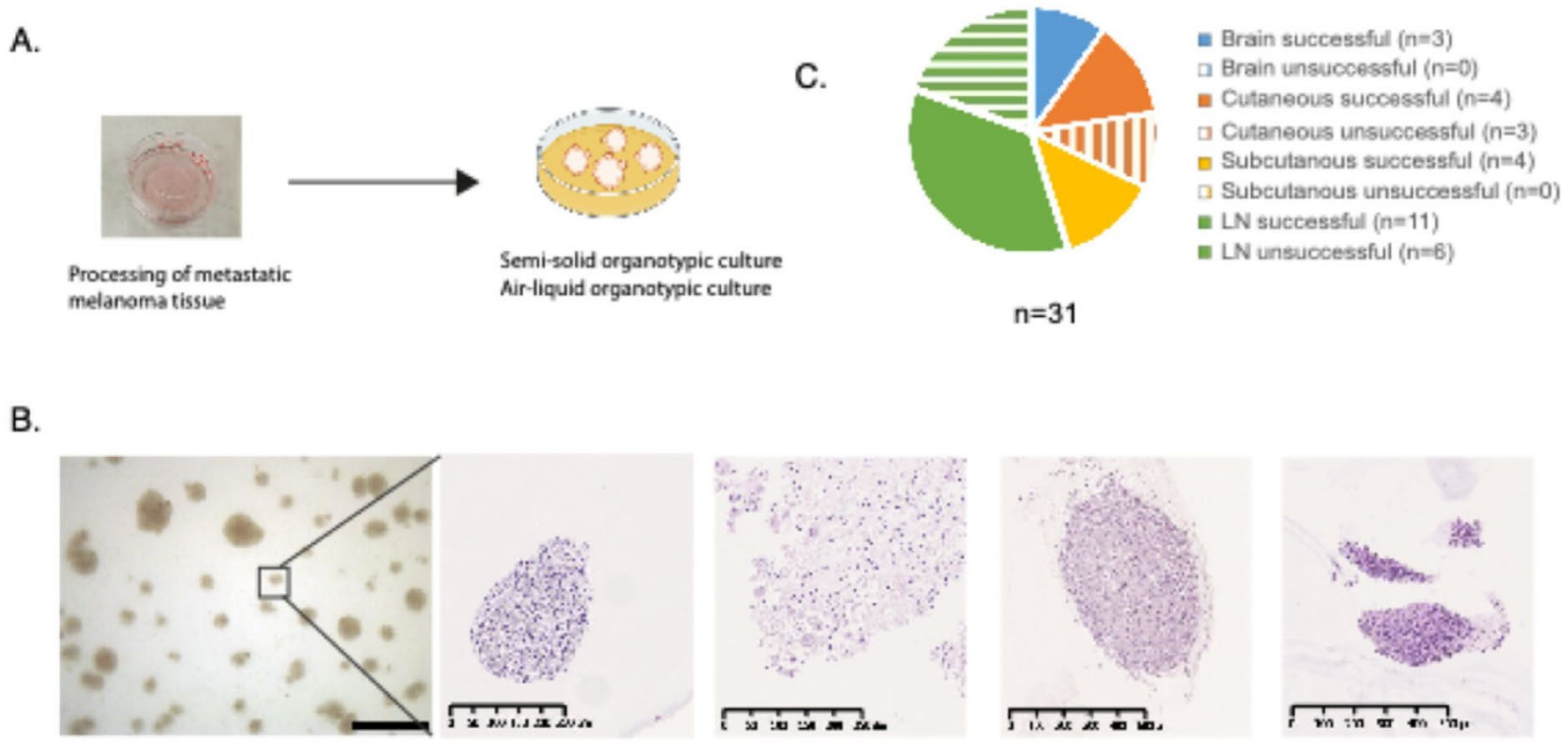
Patient-derived organotypic cultures from metastatic melanoma tissue. **A** Overall scheme of the generation of organotypic cultures. **B** Representative image of organoids growing in semi-solid media. Included are also representative hematoxylin and eosin staining images from four different organoids. **C** PDO frequency and success rate by tumor site

In this study, we aimed to develop model systems suitable for testing immunomodulatory drugs in melanoma. We obtained fresh tumor tissue at surgery from 31 patients diagnosed with metastatic melanoma. The cohort consisted of 17 lymph nodes metastases, 11 cutaneous/subcutaneous metastases, and three CNS metastases. We established patient-derived organotypic cultures from fresh tumor material with the aim of preserving a diverse tumor microenvironment. Two types of cultures were established: semi-solid patient-derived organotypic cultures [12] and air–liquid interface patient-derived organotypic cultures [13] (Fig. 1a). Semi-solid PDO cultures were considered successful upon the formation of three-dimensional spheroid structures within the first week of culture while successful air–liquid PDO cultures exhibited spheroids or dense tumor fragments that maintained their structure and integrity over time (Fig. 1b and Fig. S1**-2**). The overall success rate was 71%, corresponding to successful establishment of material from 22 out of 31 samples using at least one of the two culture methods (Fig. 1c)*.* Air–liquid PDO cultures were successfully established in 11 out of 13 samples (85%), including some samples where semi-solid PDO cultures failed. In conclusion, we have successfully generated patient-derived organotypic cultures from different metastatic sites including the brain, lymph node, and subcutaneous skin tissues.

### Baseline metastasis and baseline PDOs display genomic similarities

**Fig. 2:**
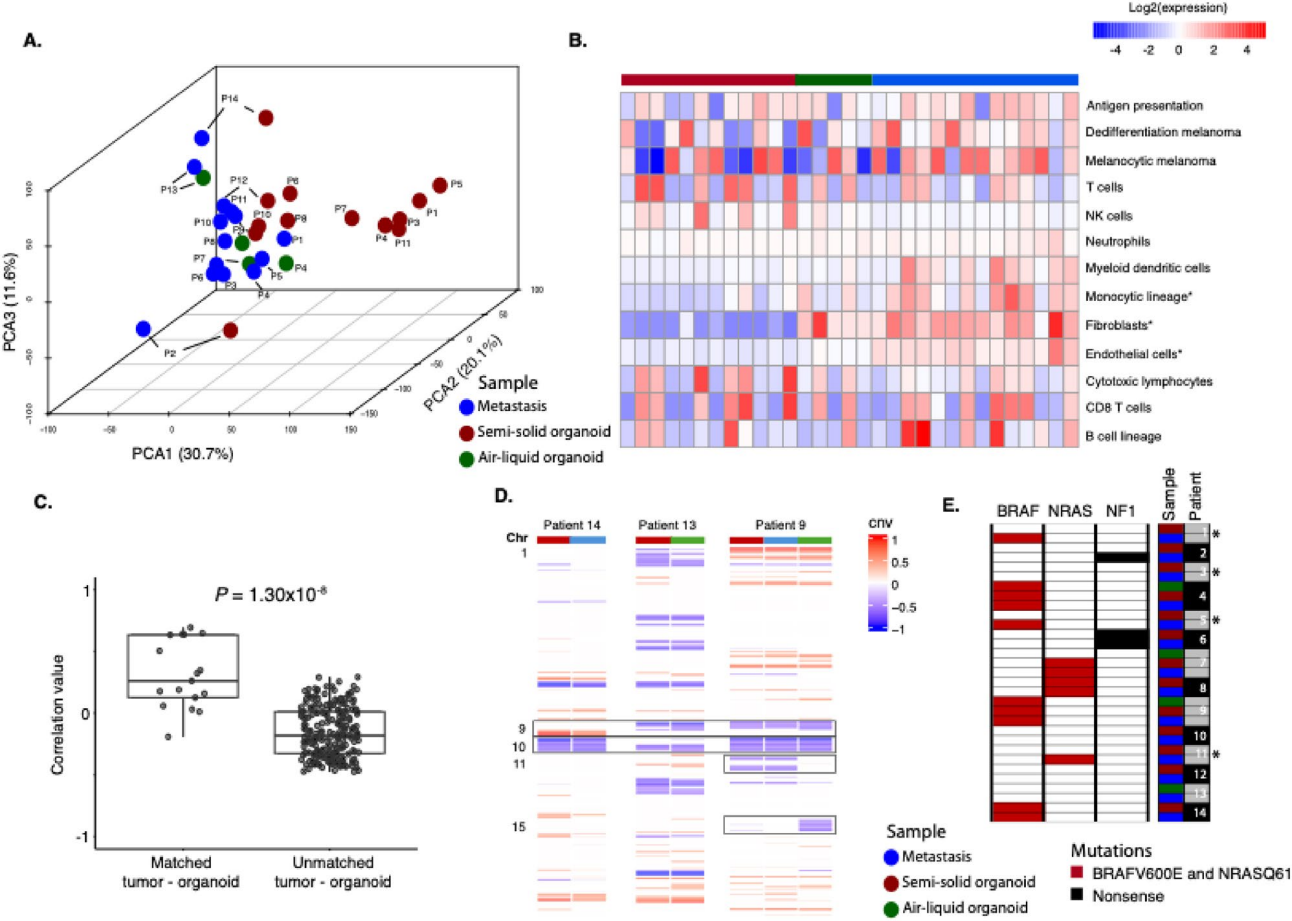
Genomic and transcriptomic properties of melanoma metastasis and matched organotypic culture. **A** Principal component analysis (PCA) of the 5000 most variable genes in metastatic tissue and matched semi-solid PDO and air–liquid PDO. Variation in percent in each PCA component is indicated. **B** Heatmap displaying gene expression values of the microenvironment and cell populations-counter (MCP) scores. In addition, gene expression scores representing antigen presentation (HLA-A, HLA-B, HLA-C, B2M, TAP1), melanocytic (SOX10, MITF, TYR, PMEL), and dedifferentiated melanoma (SOX10, NGFR, AXL) are displayed. **C** Boxplot of gene expression correlation values between matched metastatic lesion and PDO and values from unmatched metastatic lesion and PDO. **D** Representative DNA copy number profiles generated from RNA sequencing data. Chromosomes 9 and 10 are highlighted since these are recurrently lost in melanoma tumors. Chromosomes 11 and 15 show differences between metastasis and PDO. **E** Hotspot and loss of function mutations in BRAF, NRAS, and NF1 in matched metastasis and PDO. * marks semi-solid organoids from patients P1, P3, P5 and P11 in the PCA plot in A)

Next, we generated whole transcriptomic data from tumor biopsy and matched patient-derived organotypic culture using RNA sequencing. Sufficient RNA was collected from 14 PDOs and matched metastatic deposit with different clinical characteristics (Table 1). Using principal component analysis (PCA), we observed that metastasis and matched PDO were broadly similar in the transcriptional space (Fig. 2a). To identify cell-type-specific differences between the experimental settings, we calculated microenvironment and cell populations-counter (MCP) scores. While most MCP signatures showed similar expression levels, monocytic, fibroblastic, and endothelial cell signatures were elevated in baseline tumors compared to PDO (Fig. 2b). Reassuringly, T and B cell signatures were preserved during organoid formation. Next, we aimed to statistically assess each PDO was more transcriptionally similar to its corresponding metastasis than to unrelated metastases. We calculated the correlation between each PDO and all analyzed metastatic tumor lesions. Matched PDO and metastases showed significantly higher correlation values compared to unmatched pairs, confirming that transcriptional properties are preserved during organotypic culturing (*P* = 1.3 × 10^–8^, Fig. 2c). Next, we generated genome-wide copy number profiles using the RNA sequencing data. Frequent melanoma-specific copy number changes such as loss of chromosomes 9 and 10 were identified across all samples, and those were largely preserved from metastatic tumors to the organotypic cultures. Minor differences were found, such as loss of chromosome 11 in the PDO in Patient 9. In the same case, we observed loss of chromosome 15 that was not detected in the PDO. Nevertheless, the transcriptional data were supported by matched PDO and metastases displaying an overall similar DNA copy number profiles (Figs. 2d, S3). This was further supported by somatic mutations in *BRAF*, *NRAS,* and *NF1* genes that were preserved during the formation of organoids (Fig. 2e). Moreover, a set of five semi-solid PDOs clustered together in the PCA plot (Fig. 2a). Detailed analysis of DNA copy number data and hot-spot mutations revealed devoid of genetic alterations. Histological analysis using SOX10 staining further confirmed the absence of melanoma cells (Figs. S3–4). In all, these data suggest that these PDOs lack melanoma cells and thus do not represent their matched metastatic lesions.

**Table 1:** Clinical features of samples where organotypic cultures were included in the transcriptomic analyses

Clinical feature		Fraction
*Gender*	Male Female	N = 11, 79% N = 3, 21%
*BRAF mutation* *(clinical testing)*	V600E Wt NA	N = 4, 29% N = 8, 57% N = 2, 14%
*Primary tumor site*	Lower extremity Upper extremity Trunk HN Unknown primary NA	N = 2, 14% N = 1, 7% N = 1, 7% N = 4, 29% N = 5, 36% N = 1, 7%
*Primary tumor type*	SSM NM Mucosal Unknown primary	N = 7, 50% N = 1, 7% N = 1, 7% N = 5, 36%
*Primary tumor Breslow*	5.8 mm, range 2.2–14 mm	
*Metastasis*	LN SC CNS	N = 10, 71% N = 3, 21% N = 1, 7%
*Stage at metastasis*	IIIB IIIC IIID M1a M1d	N = 3, 21% N = 6, 43% N = 2, 14% N = 2, 14% N = 1, 7%
*Sampling with regard to treatment*	Pre-ICB treatment Relapse after ICB On-ICB treatment Therapy naïve NA	N = 6, 43% N = 5, 36% N = 1, 7% N = 1, 7% N = 1, 7%

ICB—immune checkpoint blockade

In conclusion, the generation of PDOs preserves important genomic properties of the original metastases.

### Cellular and histological characteristics of PDOs

**Fig. 3:**
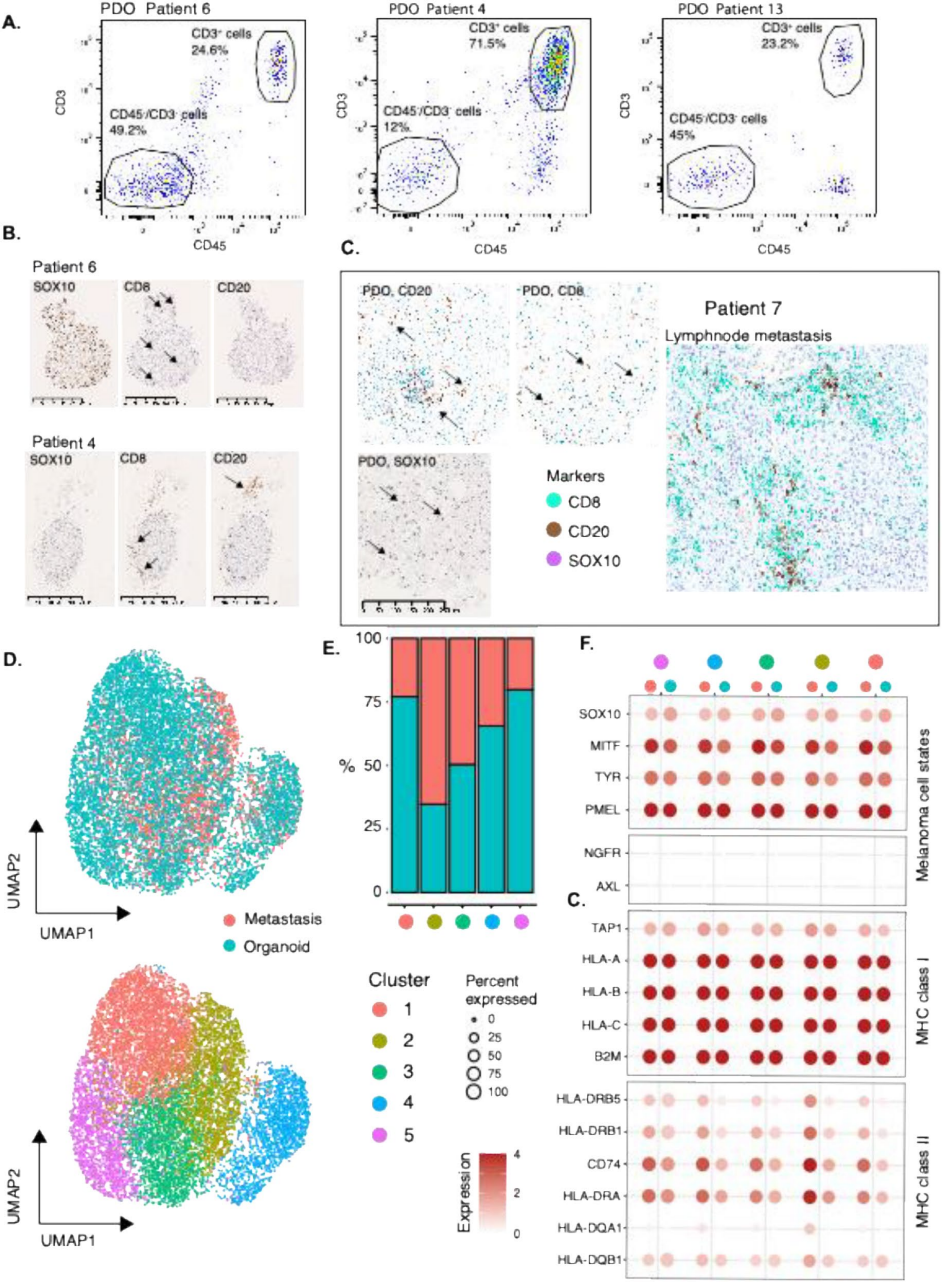
Cellular and histological characterization of organoids. **A** Representative flow cytometry analysis charts of organotypic cultures from Patients 4, 6, and 13. Antibodies against CD3 and CD45 were used. **B** Immunostaining of SOX10, CD8, and CD20 in organotypic cultures from Patients 4 and 6. **C** Immunostaining of SOX10, CD8, and CD20 in organotypic culture and matched metastasis from Patient 7. **D** UMAP plot using single-cell RNA sequencing data from organotypic culture and matched metastasis from Patient 6. **E** Barplot of sample representation in each UMAP cluster. **F** Dotplot showing expression average of melanoma related genes in each UMAP cluster. Average value of all cells belonging to metastasis and organoid separately is displayed

Next, we evaluated cellular and histological characteristics of baseline PDO and their matched metastasis. First, we used flow cytometry analysis to determine the fraction of CD3^+^/CD45^+^ T cells in the PDOs. The proportion of CD3^+^/CD45^+^ T cells varied widely, ranging from 0.5% to 92%, reflecting the heterogeneity across cultures (Fig. 3a). In addition, different proportions of CD45^+^/CD3^−^ immune cell populations were detected, suggesting the presence of immune cell types other than T cells. Secondly, we performed immunostaining of PDOs to determine immune cell localization and found a substantial heterogeneity in the presence and infiltration of CD8^+^ T cells within the organoids (Figs. 3b, S5). Notably, CD8^+^ T infiltration of the organoid was limited in the majority of PDOs. Visible CD20^+^ B cells in conjunction with an organoid were only observed in two cases (Fig. 3b). One case (Patient 7), despite massive infiltration of CD8^+^ T and CD20^+^ B cells within the organoids, SOX10^+^ melanoma cells were still present (Fig. 3c). The matched metastatic lesion was subsequently analyzed by immunostaining for immune cells presence. Consistent with organoid findings, the metastatic lesion of organoid from Patient 7 displayed extensive CD8^+^ T cell infiltration with the presence of lymphoid aggregates with predominantly CD20^+^ B cells [24] (Fig. 3c). In contrast, Patient 6 exhibited limited infiltration of CD8^+^ T cells that was similarly reflected in the corresponding organoid (Fig. S6). Clearly, not all T cells are infiltrating the actual organoid but are part of the organotypic culture as demonstrated in the flow cytometry data (Fig. 3a). Melanoma cell states are known to shift in response to microenvironmental cues [29]. To investigate this, we performed single-cell RNA sequencing (scRNAseq) on the metastatic lesion from Patient 6 and, in parallel, on tumor organoids isolated from the corresponding culture. Melanoma cells were identified based on SOX10 expression, and clustering with UMAP was used to define distinct melanoma cell states. Overall, 10.258 melanoma cells were used in the analysis. Previously, Pozniak et al. described melanoma cell states by scRNAseq [21]. To recapitulate these states, we used the geneset described in Pozniak et al. This approach identified five UMAP clusters with cluster 2 enriched for melanoma cells from the metastatic lesion and cluster 1 enriched for melanoma cells from the PDO (Fig. 3d-e). However, none of the clusters were exclusive to either metastatic lesion or PDO (Fig. 3e). Moreover, all clusters expressed both melanocytic and antigen-presenting molecules indicating only subtle changes between them and suggesting that overall melanoma cells from the PDO closely resemble those in the metastatic lesion (Fig. 3f).

In conclusion, the presence of immune cells in baseline metastatic lesion is reflected in PDOs, suggesting an intact anti-tumor immune response. Moreover, melanoma cell states are preserved in the PDOs, reflecting those of the original tumors.

### Transcriptional effect of T cell stimulation is based on clinical response to ICB

**Fig. 4:**
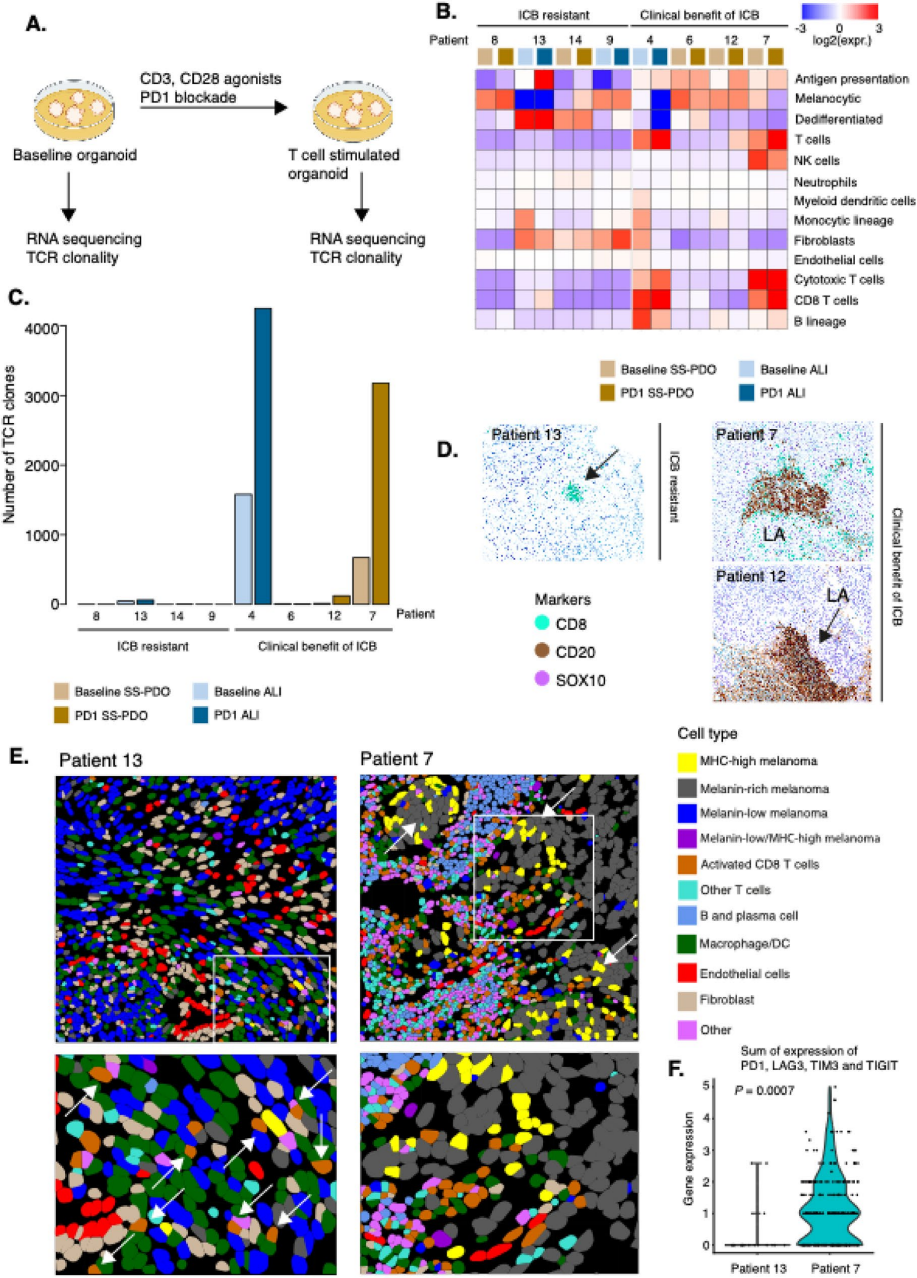
T cell stimulation in organotypic cultures and clinical benefit of immune checkpoint blockade (ICB). **A** Overall scheme of experimental setup. **B** Microenvironment and cell populations-counter (MCP) scores from baseline and PD1 therapy-treated organotypic cultures. In addition, gene expression scores representing antigen presentation (HLA-A, HLA-B, HLA-C, B2M, TAP1), melanocytic (SOX10, MITF, TYR, PMEL), and dedifferentiated melanoma (SOX10, NGFR, AXL) are displayed. Cases are grouped according to patient ICB therapy response. **C** Barplot showing number of specific T cell clones using T cell clonality analysis. Cases are grouped according to patient ICB therapy response. **D** Representative immunostaining of three cases. Arrows highlight T cells in tumors. LA refers to lymphoid aggregates. **E** Spatial transcript profiling of two melanoma metastases from Patients 7 and 13, respectively. In Patient 7, arrows indicate antigen-presenting melanoma cells. In Patient 13, arrows indicate CD8.^+^ T cells. Zoom-in regions are marked by a square. **F** Violin plot of a composite gene expression score of checkpoint molecules (PDCD1, LAG3, TIGIT, HAVCR2) in CD8 + T cells from Patients 13 (n = 20) and 7 (n = 261)

To test the relevance of PDO for evaluating anti-cancer immune responses, we used four PDOs derived from patients who experienced clinical benefit from PD1 blockade. As controls, we used four PDOs derived from patients who relapsed following either single-agent PD1 blockade or combined PD1/CTLA4 therapy. To determine the early effects of T cell stimulation, we treated PDOs with CD3 and CD28 agonists in combination with a PD1 blocker for 1 week. PDOs were processed for RNA sequencing pre-treatment and one week of treatment (Fig. 4a). We then applied MCP-counter to infer presence and abundance of cell types within the cultures. Broadly, baseline PDOs from patients having clinical benefit to ICB showed higher expression scores for antigen presentation, while those from ICB-resistant metastases exhibited increased fibroblastic signatures. Although PDOs from patients with clinical benefit from ICB showed higher T cell-related gene expression scores, extensive heterogeneity was observed (Fig. 4b). Additional melanocytic signatures described in Hu et al. [30] were included to determine how and if melanoma cells change under T cell stimulation (Fig. S7). Next, we determined T cell receptor clonality (TCR) and found a higher frequency of T cell clones in baseline PDOs from ICB responders. In three of these patients, T cell stimulation led to clonal expansion, whereas no such expansion was observed in cultures from non-responders (Fig. 4c). Overall, T cell clone abundance was significantly correlated with T cell gene expression signature (Spearman = 0.804, P = 5.82 × 10^–5^, Fig. S8). Moreover, PDOs from responders showed increased T cell diversity following T cell activation (Fig. S9). Histology analyses of the matched metastatic lesions from Patients 4, 7, and 12 identified distinct regions of immune clusters resembling tertiary lymphoid structures (Fig. 4d) suggesting that such melanomas with benefit of ICB are susceptible to T cell reinvigoration. Patients 4 and 12 both received adjuvant PD1 blockade and were recurrence-free after > 1 year. Patient 7 in the cohort received adjuvant anti-PD1/LAG3 therapy, which was halted due to toxicity. A recurrence was detected after almost a year after surgery. The patient subsequently received anti-PD1/CTLA4 therapy, which was discontinued after two cycles. At the most recent follow-up (< 6 months from treatment pause), no disease progression had been observed. In contrast, histological analysis of metastatic lesion from patient 13 displayed prominent CD8^+^ T cell presence, however, spatially localized in clusters (Fig. 4d). The other three ICB-resistant cases all had very few infiltrating CD8^+^ T cells (Fig. S10). Next, we wanted to understand why there was a T cell expansion in patients with clinical benefit of ICB while this was not observed in samples from patients resistant to ICB. Thus, we leveraged single-cell spatial transcriptomics of two metastatic lesions from Patients 7 and 13. In Patient 7, we observed a wide range of different immune cells including activated CD8^+^ T cells, cells of the monocytic lineage, B cells and other T cells (Fig. 4e). We also found a significant proportion of melanoma cells expressing MHC class I suggesting that they are actively presenting antigens. Such cells were frequently located in proximity to immune cells while melanoma cells spatially located distant from immune cells had a decreased expression of MHC class I (Fig. 4e). In contrast, we generated the same type of data from Patient 13 that had relapsed on adjuvant anti-PD1 therapy but still had a substantial number of T cells which did not show clonal expansion ex vivo (Fig. 4c). We found that this melanoma lacked tumor cells expressing MHC class I molecules and phenotypically such melanoma cells also had decreased melanocytic transcriptional properties. We also observed a massive infiltration of macrophages but also CD8^+^ T cells (Fig. 4e). When analyzing the transcriptional phenotypes of CD8^+^ T cells in these two melanomas, we found that CD8^+^ T cells in Patient 7, who had benefit of ICB therapy, had expression of checkpoint molecules such as PD1, LAG3, TIM3, and TIGIT, while CD8^+^ T cells from Patient 13 completely lacked expression of such checkpoint/activation molecules (P = 0.0007, Fig. 4f).

Collectively, these findings indicate that T cells from patients who benefit of ICB are capable of expanding ex vivo and display transcriptional profiles consistent with activation and checkpoint engagement.

### T cell stimulation in PDOs from therapy naïve melanoma tissue confers a distinct transcriptional profile predictive of ICB clinical response

**Fig. 5:**
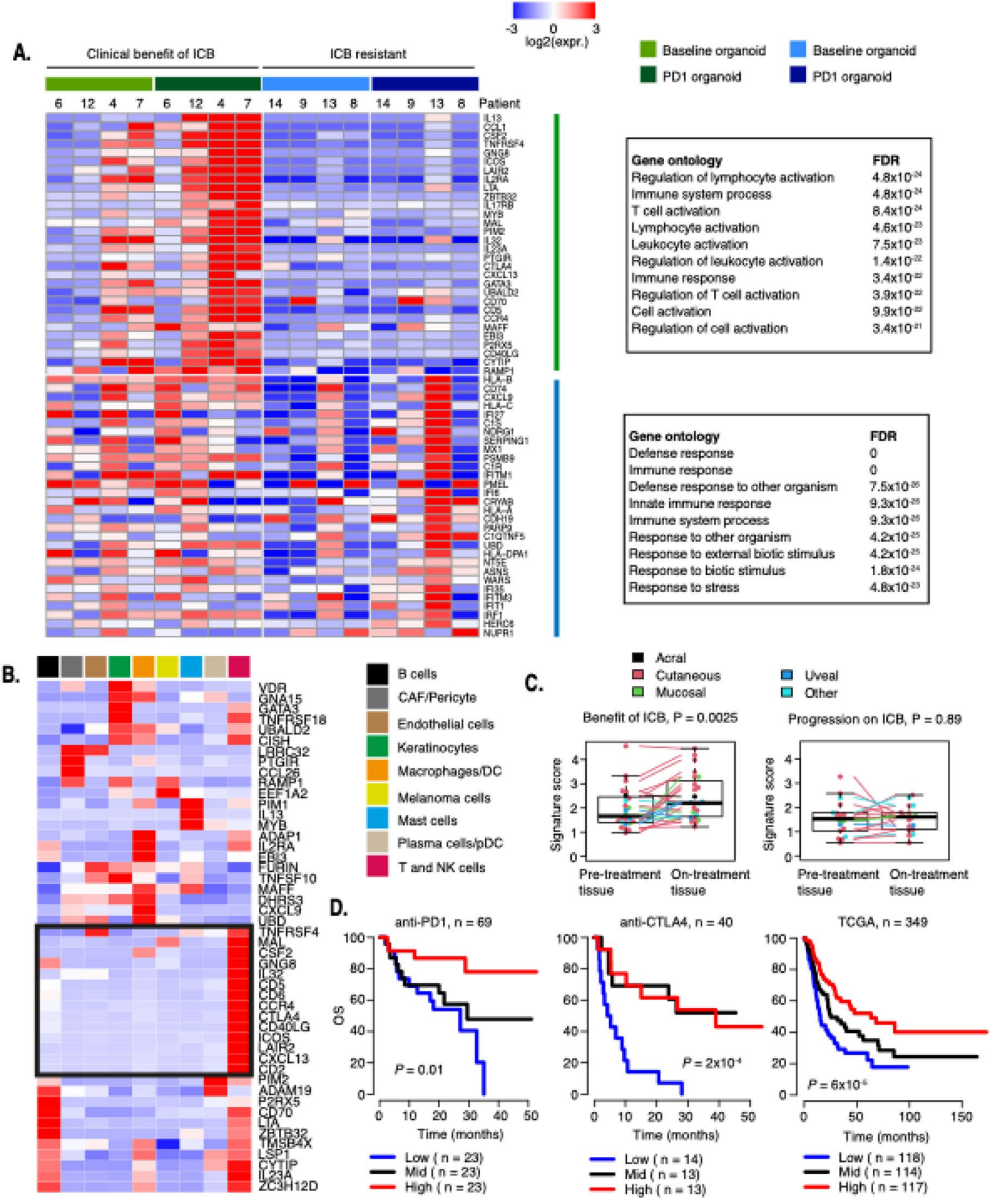
ICB-induced transcriptional profiles in organotypic cultures. **A** Heatmap of gene expression changes from baseline to PD1 blockade-treated organotypic culture in cases from ICB responders (green bar) and ICB-resistant cases (blue bar). Gene ontology analysis of each gene set is displayed. **B** Heatmap of scaled mean expression of genes upregulated in organotypic cultures from patients with benefit of ICB. Single-cell RNA sequencing data were retrieved from Pozniak et al. [21]. The 14 genes exclusively expressed in T cells are indicated. **C** Gene expression score of the 14 T cell specific genes was used on bulk RNA sequencing data of matched pre-/on-treatment samples (n = 42) from Riaz et al. [25]. **D** Kaplan–Meier progression-free survival (PFS) and overall survival (OS) analysis. Stratification is based on the 14-gene T cell specific signature score on bulk RNA sequencing data of metastatic melanoma patients (n = 69) from Gide et al. [28], (n = 40) van Allen et al. [26], and (n = 349) TCGA. [31]

We next determined the gene programs upregulated by T cell stimulation ex vivo in PDOs from patients with clinical benefit from ICB. Intriguingly, we observed strong induction of genes related to T cell activation including, CCL1, OX40, ICOS, CTLA4, and CXCL13 (Fig. 5a). Consistent with the T cell clonality results, Patients 4 and 7 displayed the strongest effect on gene expression changes. However, similar transcriptional responses were also observed in the other two PDOs from patients who had benefited from ICB. In these ICB responder cases, gene ontology analysis revealed enrichment of general immune system processes such as lymphocyte and T cell activation. Intriguingly, these genes remained unaltered in T cell-stimulated PDOs derived from patients who previously had relapsed on ICB (Fig. 5a). Next, we investigated genes upregulated in PDOs from patients who had relapsed on ICB including Patient 13 whose baseline culture contained a substantial number of T cells (Fig. 4c). The genes displaying the highest fold change between baseline and stimulated PDOs from ICB-resistant tissue were enriched for innate immune response genes. Notably, this transcriptional increase was predominantly found in a single patient, Patient 13. This patient relapsed 9 months after adjuvant anti-PD1 therapy and underwent surgical resection of a subcutaneous lesion prior to initiating dual PD1/CTLA4 blockade. At eight-month follow-up, the patient remained recurrence-free.

Given the consistent upregulation of a transcriptional program in PDOs treated with T cell stimulatory molecules derived from patients with clinical benefit from ICB, we wanted to explore this in detail. Analysis of single-cell RNA sequencing data from melanoma metastases [21] revealed that 14 of the upregulated genes were exclusively expressed in T cells (Fig. 5b). To determine the predictive value of these 14 T cell-specific genes, we created a composite score and applied this on bulk RNA sequencing data from two independent datasets [25, 28]. In the Riaz et al. [25], RNA sequencing data on pre- and on-treatment biopsies from 42 metastatic melanoma patients were available. Confirming our ex vivo results, we found that the 14-gene T cell signature was increased in on-treatment biopsies from patients with stable disease, partial response, or complete response (Fig. 5c). Such increase in gene expression signature was not observed in patients with progressive disease on ICB (Fig. 5c). Finally, we applied the 14-gene T cell signature on bulk RNA sequencing data from 458 melanoma patients. These included data from 69 melanoma patients receiving anti-PD1 or anti-PD1/CTLA4 therapy [28], 40 melanoma patients receiving anti-CTLA4 [26], and the Cancer Genome Atlas cohort that predominantly includes patients not treated with ICB therapy [31]. Stratifying patients into tertiles based on the T cell gene expression score displayed significant difference in progression-free and overall survival (Fig. 5d, P = 0.01) in patients with highest T cell gene expression score.

In conclusion, transcriptomic signature derived from ex vivo melanoma models can be utilized to predict ICB clinical response.

## Discussion

In this study, we generated and validated PDO systems from metastatic melanoma. These platforms serve as physiologically relevant models to evaluate immunomodulatory therapies. Utilizing fresh tumor tissues from 31 patients, we tested two culture approaches designed to preserve the cellular heterogeneity and spatial organization of the tumor microenvironment (TME). The overall success rate was 71%, with the air–liquid PDOs showing a higher establishment rate (85%). Notably, the latter succeeded even in cases where semi-solid PDOs failed, indicating the robustness of this model for capturing TME complexity ex vivo. These results corroborate previous efforts to develop organotypic culture systems for human cancers [13].

In our study, transcriptomic profiles of the PDOs closely matched their parental metastatic lesions. Although microenvironment-associated gene signatures such as monocytic, fibroblastic, and endothelial components were decreased in PDOs, critical immune cell lineages, including T and B cells, were preserved. These findings are consistent with the notion that organoid cultures can partially retain the TME, especially lymphoid components, which play pivotal roles in mediating response to immunotherapy [32]. Furthermore, matched tumor-PDO comparisons revealed significantly higher transcriptional correlation than unmatched pairs, confirming that PDO largely conserves tumor-intrinsic transcriptional programs. Copy number variation analysis from RNA-seq data also revealed conserved melanoma-specific alterations, including frequent losses of chromosomes 9 and 10, which are well documented in melanoma pathogenesis [31]. Genomic heterogeneity as measured by subclonal mutations has demonstrated to correlate with response to ICB [33, 34]. However, in our study we did not address subclonal mutational and copy number changes because RNA sequencing-based mutational calling is not sufficiently accurate for such analyses. Moreover, single-cell RNA sequencing (scRNAseq) of melanoma cells from matched tumor and PDO identified overlapping transcriptional states, characterized by shared expression of melanocytic markers and antigen presentation genes. This suggests that key tumor-intrinsic programs are preserved ex vivo. Further confirming that molecular states are preserved during PDO formation immunostaining revealed variable infiltration of CD8^+^ T cells in the organoids. In one case (Patient 7), substantial infiltration was observed and reflected the matched metastatic lesion. In contrast, Patient 6 exhibited minimal immune infiltration in both the PDO and tumor. These findings support the reliability of PDOs in reflecting the immune landscape of the original tissue, consistent with previous reports in melanoma organoids [10].

To investigate the utility of PDOs in predicting response to ICB therapies, we stimulated organotypic cultures from patients with known clinical benefit from PD1-based therapy, as well as from patients who had relapsed after ICB, using T cell agonists and PD1 blockade. Post-treatment transcriptional data revealed significant upregulation of T cell activation signatures (e.g., CCL1, OX40, ICOS, CTLA4, CXCL13) in PDOs from ICB responders. In contrast, these changes were absent in PDOs derived from ICB-resistant cases. The chemokine CXCL13, for instance, is expressed by activated T cells and other stromal cells and plays a central role to support TLS formation. Previous work has shown its predictive value for ICB responses in melanoma [35]. Clonotype analysis revealed T cell expansion and increased TCR diversity in responder PDOs, but not in resistant cases, suggesting that the ex vivo models recapitulate in vivo immune responsiveness. Expansion of T cells was associated with increased MCP T cell expression score. Previous data have described that immune gene expression scores are independent of tumor mutational burden [36] thus implicating that TMB is independent of T cell expansion. Instead, the PDOs showing the strongest T cell expansion following PD1 blockade were derived from matched metastatic lesions harboring lymphoid aggregates that could reflect tertiary lymphoid structures (TLS). TLS are linked to improved outcomes in melanoma and other cancers due to their role in sustaining localized anti-tumor immune responses [24, 37]. To explore the mechanism underlying these observations, we performed single-cell spatial transcriptomics on matched tumors samples. In ICB responders, melanoma cells expressing MHC class I were found in proximity to activated CD8^+^ T cells, consistent with effective antigen presentation and T cell priming. Conversely, in ICB-resistant cases, such as Patient 13, melanoma cells lacked MHC class I expression and melanocytic markers and CD8^+^ T cells failed to express activation or checkpoint markers (e.g., PD1, LAG3, TIM3), suggesting a dysfunctional immune microenvironment. These observations are supported by earlier studies in that have linked MHC class I loss and antigen presentation defects with ICB resistance [38–40].

Using the generated bulk RNA sequencing data from stimulated PDOs of ICB responders, we derived a 14-gene T cell activation signature. This signature was validated in independent clinical datasets [25, 28]. These findings suggest that PDOs, when used in conjunction with T cell stimulation assays, can serve as a functional readout of a patient’s capacity to mount an immune response and may aid in stratifying patients for immunotherapy.

In conclusion, our findings establish that patient-derived organotypic cultures from metastatic melanoma retain key genomic, cellular, and immunological features of the original tumor. The ability to simulate T cell activation and monitor clonal expansion and transcriptional changes ex vivo provides a promising avenue for biomarker discovery and personalized immunotherapy testing.

## Supplementary Information

Below is the link to the electronic supplementary material.

Supplementary file1 (PDF 1849 KB)

## Data Availability

Requests for information and resources should be directed to and will be fulfilled by the lead contact, Göran Jönsson (goran_b.jonsson@med.lu.se). Raw sequencing data are regarded as personal information, and by Swedish law, they cannot be made publicly accessible. However, information and mechanisms for data access can be obtained by contacting the corresponding author.
