# Supplementary material for: Modeling anti-tumor immune responses using patient-derived melanoma organoids
Source: Cancer Immunol Immunother. 2025 Dec 23;75(1):24. doi: 10.1007/s00262-025-04269-9 (PMC12728156; doi:10.1007/s00262-025-04269-9)
Supplement: Supplementary file 1 — Supplementary file1 (PDF 1849 KB) [file 262_2025_4269_MOESM1_ESM.pdf]

Supplementary information

Modelling anti-tumor immune responses using patient-derived melanoma organoids

Kamila Kaminska[1,2], Bengt Phung[1,2], Jacob Karlström[1,2], Martin Lauss[1,2], Katja Harbst[1,2], Teresa Svensson[3,4], Kristian Pietras[2,5], Kari Nielsen[2,4,6,7,8], Ana Carneiro[1,2,3,4], Henrik Ekedahl[2,3,4], Karolin Isaksson[2,9,10], Göran Jönsson[1,2,*]

1. Division of Oncology, Department of Clinical Science, Faculty of Medicine, Lund University, Lund, Sweden
2. Lund University Cancer Center
3. Department of Oncology, Skåne University Hospital, Lund, Sweden
4. Skåne University Hospital Comprehensive Cancer Center, Lund, Sweden
5. Division of Translational Cancer Research, Department of Laboratory Medicine, Lund University, Lund, Sweden
6. Department of Dermatology, Helsingborg General Hospital, Helsingborg, Sweden
7. Department of Dermatology, Skåne University Hospital, Lund, Sweden
8. Division of Dermatology, Department of Clinical Science, Faculty of Medicine, Lund University, Lund, Sweden
9. Department of Surgery, Skåne University Hopsital, Kristianstad, Sweden
10. Division of Surgery, Department of Clinical Science, Faculty of Medicine, Lund University, Lund, Sweden

Supplementary methods

Patient samples

Tumor material used to establish patient derived organotypic cultures was acquired from patients undergoing surgical resection for metastatic malignant melanoma at Skåne University Hospital, Kristianstad General Hospital and Helsingborg General Hospital included in the BioMEL study [1] (ClinicalTrials.gov ID NCT05446155). All patients undergoing metastatic melanoma surgery (years 2021-2023) from whom tissue was obtained provided written informed consent for the collection of tissue and matched normal blood samples for research as approved by the local ethical board (Lund University Ethical Review Board, Dnr. 2013/101). The study adhered to the declaration of Helsinki.

Data availability

Raw sequencing data are regarded as personal information, and by Swedish law, they cannot be made publicly accessible. However, information and mechanisms for data access can be obtained by contacting the corresponding author.

Tissue preparation and cryopreservation

Immediately after a surgery available tissue was kept at 4 deg C in ADMEM/F12 media (Thermo Fisher, cat. 12634028) supplemented with 100 µg/ml primocin (InvivoGen, cat. ant-pm-2), 2,5 µmol thiazovivin (Selleckchem, cat. S1459, vol. conc.-dependent), 100 nmol SB431542 (Selleckchem, cat. S1067, vol. conc.-dependent), 4 µmol CHIR99021 (Selleckchem, cat. S1263 vol. conc.-dependent) and 10 µmol Y27632 (Selleckchem, cat. S6390 vol. conc.-dependent), referred to as processing media. Tissue biopsies were processed fresh, as soon as possible, usually within 1-2 hours from the surgical procedure. When tissue could not be processed immediately it was stored at 4 deg C for no longer than 24 hours. The small molecule inhibitors were added for anti-apoptotic and pro-proliferative effects as described[2].

Tissue was minced finely on ice using sterile scissors until obtaining fragments of about 1 mm^3 in size and washed with processing media. If blood was visible, red blood cell (RBC) lysis was performed for 5 minutes on ice using RBC lysis buffer (0,15 M NH_4Cl (Sigma Aldrich, cat. A9434), 12 mM $NaHCO_3$ (Sigma Aldrich, cat. S5761), 0,1 mM EDTA (Sigma Aldrich, cat. E6758)) followed by additional wash with processing media [3]. Minced tumor material was divided between fresh organotypic cultures and cryopreservation in stem cell freezing media (ATCC, cat, ACS-3020).

Patient derived organotypic cultures

Two types of patient-derived organotypic (PDO) cultures were set up from fresh tumor material Semi-solid PDO cultures modified from Vilgelm et al.[4] and air-liquid interface (ALI) cultures as in Neal et al.[5] with modifications. The complete culture media used for both types of cultures consisted of: ADMEM/F12 (Thermo Fisher, cat. 11320033) supplemented with 50% Wnt3a, R-spondin 3, noggin-conditioned media (L-WRN, ATCC), 5% FBS (Thermo Fisher, cat. A5670701), 2 mM UltraGlutamine (Lonza, cat. BEBP17-605E), 10 mM nicotinamide (Sigma, cat. N0636), 1 mM N-acetyl-L-cysteine (Sigma, cat. A9165), 1x B-27 without vitamin A (Thermo Fisher, cat. 12587010), 0,5 µM A83-01 (Tocris, cat. 2939), 10 nM Gastrin (Sigma Aldrich, SCP0152), 1000 IU human recombinant IL-2 (R&D Systems, 202-cat. IL-050), 1x Human Melanocyte Growth Supplement (Thermo Fisher, cat. S0025) and 100 µg/ml primocin (InvivoGen, cat. ant-pm-2).

Semi-solid PDO cultures

Freshly minced tissue was filtered through 100 µm cell strainer and the flow-through solution consisting of single cells and small cell clusters was collected for plating. Remaining larger tissue fragments were disposed. Below-100 µm fraction was resuspended in complete culture media with 10% Matrigel (Corning, Basement Membrane Matrix, LDEV-free, cat. 354234). Cell suspension was plated at 500 µl/well in 24-well ultra-low attachment plates (Corning) and placed in a cell incubator at 37 deg C. In the first 3-4 days of culture, media was supplemented with 2,5 µmol thiazovivin (Selleckchem, cat. S1459, vol. conc.-dependent), 100 nmol SB431542 (Selleckchem, cat. S1067, vol. conc.-dependent), 4 µmol CHIR99021 (Selleckchem, cat. S1263 vol. conc.-dependent) and 10 µmol Y27632 (Selleckchem, cat. S6390 vol. conc.-dependent). To replenish the media and compensate for media loss due to evaporation, 200 µl of complete culture media with 10% Matrigel was added drop-wise to each well weekly.

Air-liquid interface (ALI) cultures

Prior to plating tumor tissue, 800 µl of base collagen layer was added into a transwell insert (30 mm diameter, 0,4 µm pores Millicell-PCF, Millipore, cat. PIHP03050) placed in a 60-mm culture plate to form the double dish air-liquid culture system. Basal collagen matrix contained 4 mg/ml collagen (Cultrex 3D Culture Matrix Rat Collagen I, BioTechne, cat. 3440-100-01) neutralized with collagen reconstitution buffer (2,2 g $NaHCO_3$ (Sigma-Aldrich, cat. S5761) in 100 mL of 0,05 M NaOH (Sigma-Aldrich, cat. 567530) and 200 mM HEPES (Thermo Fisher, cat. **15630056**)) and complete culture media. The gel was solidified at 37 deg C for 30 min in a cell incubator. Freshly minced tissue was resuspended in 1ml collagen-matrigel matrix (2,5 mg/ml collagen neutralized with collagen reconstitution buffer, 10% Matrigel (Corning, cat. 354234) and complete culture media) and layered on top of a pre-solidified base collagen layer

in a transwell. The gel was solidified at 37 deg C in a cell incubator. Mixture of collagen and Matrigel was used to reduce the number of cells migrating out from the tumor fragments as in (Voabil et al., 2021). ALI cultures were fed with 1-2 ml complete culture media in the outer dish, changed every 2-3 days. In the first 3-4 days of culture, the media was supplemented with 2,5 μmol thiazovivin (Selleckchem, cat. S1459, vol. conc.-dependent), 100 nmol SB431542 (Selleckchem, cat. S1067, vol. conc.-dependent), 4 μmol CHIR99021 (Selleckchem, cat. S1263 vol. conc.-dependent) and 10 μmol Y27632 (Selleckchem, cat. S6390 vol. conc.-dependent).

T cell stimulation in patient derived organotypic cultures

Organotypic cultures established as described above were recovered from the culture matrix and replated for the treatment. Both types of cultures were treated for 1 week with 10 μg/ml nivolumab (Selleckchem, cat. A2002, vol. conc.-dependent) in the presence of 2 μg/ml anti-CD3 (clone HIT3a, BioLegend, cat. 300302, vol. conc.-dependent) and 2 μg/ml anti-CD28 (clone CD28.2, BioLegend, cat. 302901, vol. conc.-dependent) antibodies. Treatment was refreshed twice.

Baseline (or Day 0) and treated samples were collected for bulk RNA sequencing. Additionally, organotypic cultures were collected for IHC staining when sufficient material was available. Sufficient RNA and PDO tissue from patients with treatment information were available for eight patients out of the 14 in Figure 2a.

Bulk RNA sequencing

PDO cultures recovered from the matrix and original tumors were processed for RNA and DNA extraction with AllPrep DNA/RNA Mini Kit (Qiagen, cat. 80204). In total, sufficient RNA was retrieved from 14 out of 22 patients. Sequencing libraries were prepared using TruSeq Stranded mRNA Library Prep kit (Illumina, cat 20020594), pooled and sequenced in a 2x150 bp paired-end setup (Illumina, cat. 20028314) on NovaSeq 6000 (Illimuna). Bulk RNA sequencing data were processed using HISAT and Stringtie (PMID: 27560171) to obtain fragments per kilobase of transcript per million mapped reads (FPKM) values. The data were reduced to protein-coding genes, quantile-normalized and log-transformed as log2 (data + 1). The data were generated at two distinct time points, however no batch effect was observed, using the R package swamp[6].

Mutation calling and copy number variation analysis using RNA sequencing data

Variants were called from hisat bam files using VarDict v.1.8.2 [7] with options: minimum allele frequency 10%, strand bias filter on. Variants were called in the following genes: *BRAF*, *NRAS*, *NF1*. The resulting variants were annotated with Annovar [8].

Copy number alterations were inferred using InferCNV (version 1.18.1). An InfercnvObject was constructed from 71 bulk RNA-seq organoid samples pooled together with 15 bulk RNA-seq samples from benign nevi, which were designated as control samples. The analysis was performed using the infercnv::run function with the following parameters: cutoff = 5, noise_filter = 0.3, and denoise = TRUE. The resulting expression matrix was log2 transformed, and genes with absolute expression values ≤ 0.01 in ≥ 50% of samples were excluded.

T cell receptor analysis from bulk RNAseq data

MiXCR[9] version 3.0.3 was applied to paired-end bulk RNAseq fastq files with the following options: mixcr analyze shotgun --species hs --starting-material rna --only-productive. The clonotypes that had count=1 were discarded. VDJtools version 1.2.1 was run on MiXCR output[10].

Immunostaining

PDO cultures were recovered from the culture matrix and fixed for 1 hour at room temperature in 10% neutral buffered formalin solution (Sigma, cat. HT501128, vol. sample-dependent), followed by a DPBS (Thermo Scientific, cat. 14190144, vol. sample-dependent) wash and sample storage at 4 deg C with added eosin (10 µl/1 ml, 0,5% Eosin Y-solution, Sigma-Aldrich, cat. 1098441000, vol. sample-dependent), prior to embedding in Richard-Allan Scientific HistoGel (Thermo Scientific, cat. PP-4030-500, vol. sample-dependent) and subsequent dehydration and preparation of FFPE block. Sections were taken and stained for SOX10 (BioCare Medical, clone BC34; 1:60, vol. 100 µl), CD45 (DAKO, clone 2B11 + PD7/26; 1:100, vol. 100 µl), CD20 (Roche, clone L26, vol. 100 µl), CD8 (Cell Marque Sigma-Aldrich, clone C8/144B; 1:100, vol. 100 µl) and B2M (Atlas Antibodies, clone HPA006361; 1:100, vol. 100 µl). Immunoslides were scanned with NanoZoomer S60 (Hamamatsu) and processed with NDP.view2.8.24 (Hamamatsu Photonics K.K.). A fragment of original tumor tissue from each patient was fixed in 10% neutral buffered formalin solution (Sigma, cat. HT501128, vol. sample-dependent) for 24 hours and processed according to standard dehydration protocol and embedded in paraffin blocks. Tissue sections (3 µm) were baked for 1 hour at 65 °C and were subjected to deparaffinization and immunohistochemistry multiplex chromogenic staining in Roche's automatic samples preparation system (Ventana Discovery Ultra) in the following steps: 1. Slides were deparaffineized using EZ Prep (Roche Diagnostics, cat. 05279771001) at 70 °C for 8 minutes. 2. Antigen retrieval was performed with Cell Conditioning 1 (CC1) (Roche Diagnostics, cat. 06414575001) at 95 °C for 64 minutes. 3. Endogenous enzyme activity was blocked using Inhibitor CM (Roche Diagnostics, cat. 07017944001, vol. 100 µl) at 37 °C for 4 minutes. 4-7. For each staining cycle, slides were incubated with a primary antibody (vol. 100 µl), followed by 100 µl of HRP-conjugated secondary antibody (Roche Discovery

OmniMap anti-Rabbit, cat. 05269679001 or anti-Mouse HRP, cat. 05269652001) at 37 °C for 16 minutes. A chromogenic substrate was then applied (DISCOVERY Green (cat. 08478295001, vol. 100 µl per reagent), Purple (cat. 07053983001, vol. 100 µl per reagent), or ChromoMap DAB (cat. 05266645001, vol. 100 µl per reagent); Roche Diagnostics) and incubated at 37 °C for 16 minutes. Antibodies were stripped between cycles using Cell Conditioning 2 (CC2, Roche Diagnostics, cat. 05279798001) at 100 °C for 8 minutes. 8. Slides were counterstained with Hematoxylin (Roche Diagnostics, cat. 05277965001, vol. 100 µl) at 37 °C for 4 minutes and incubated in Bluing Reagent (Roche Diagnostics, cat. 05266769001, vol. 100 µl) for 8 minutes at 37 °C.
SOX10 (BioCare Medical, clone BC34; 1:100, cat. 3099, vol. 100 µl), CD20 (Roche, clone L26, cat. 05267099001, vol. 100 µl) and CD3 (Roche, clone 2GV6, cat. 05278422001, vol. 100 µl) /CD8 (Cell Marque Sigma-Aldrich, clone C8/144B; 1:100, cat. 108M-95, vol. 100 µl)

Flow cytometry analysis

PDO cultures from five patients were recovered from culture matrix as described above and further dissociated into single cell suspension with Liberase TL (Roche; final concentration 50 µg/ml, cat. 05401020001, vol. sample-dependent) for 30 min at 37 deg C for ALI cultures or Accutase (Sigma, A6964, vol. sample-dependent) for 20 min at 37 deg C for PDO semi-solid cultures. After washing with DPBS, cells were labelled with LIVE/DEAD Fixable Orange (Thermo Scientific, cat. L34985) or Far Red Viability Kit (Thermo Scientific, cat. L34974), followed by incubation with FC blocking reagent (Miltenyi Biotec, cat. 130-059-901) and antibodies cocktail in FACS buffer (1% FBS in DPBS): anti-CD45-APC-Vio®770 (clone 5B1; Miltenyi, cat. 130-113-115), anti-CD3-PE (clone BW264/56; Miltenyi, cat . 130-113-129) and anti-CD19-FITC (clone HIB19; BD, cat. 555412). Samples were analyzed on FACSMelody Cell Sorter (BD Biosciences), data were analyzed with FlowJo v10.9.0.

Single cell RNA sequencing

We generated scRNAseq data from four tumor sample and semi-solid PDO. Samples were gently thawed and dissociated using Dri Tumor & Tissue Dissociation Reagent (BD Horizon, cat. 661563, vol. sample-dependent) according to manufacturer's protocol, with digestion incubation times up to 1 h. Dead cells were removed using Dead Cell Removal Kit (Miltenyi Biotech, cat. 130-090-101, vol. sample-dependent) prior to processing the remaining single cell suspension using Chromium Next GEM Single Cell 3' Kit (10x Genomics, cat. 1000269) with Chromium Next GEM Chip G (10x Genomics, cat. 1000127) with Dual Index Kit TT Set A sample barcodes (10x Genomics, cat. 1000215) according to manufacturer's recommendations. Libraries were sequenced on NovaSeq6000 (Illumina) with read length settings 28-10-10-90 as per 10x Genomics User Guide. The h5 files were processed and

merged using the R package Seurat 4.0.182, the data were reduced to protein-coding genes, translational (RPS/RPL) and mitochondrial genes (MT-), and genes which a maximum count < = 4 were removed. Cells with less than 500 expressed genes were removed. The data were normalized using SCTransform, counts that were zero before transformation were set back to zero, and data were log-transformed as log2(data+1). Cells expressing the SOX10 gene as well as not expressing the genes PTPRC, MZB1, and MS4A1 were used in downstream analyses. Harmony embeddings were calculated from the principal components, correcting the different lab protocols[11]. The data were visualized using UMAP on the top 6 harmony embeddings and clusters were identified using FindNeighbors (using top 6 harmony embeddings, k = 20) and FindClusters (Louvain algorithm, Resolution=0.3) functions of Seurat.

Single cell spatial transcriptomics

Single cell spatial transcriptomic data were obtained using the CosMx platform (Nanostring, Seattle, WA) as described previously[12]. Stromal cells were distinguished as endothelial cells and fibroblasts. Tumor cells with B2M log-transformed expression >=3 were considered "MHC-I high", tumor cells without any expression of MITF, TYR, MLANA and PMEL were considered "Melanin-low", and else were considered "Melanin-high".

External single cell RNA sequencing data

Single cell RNA sequencing data from Pozniak et al.[13] were downloaded as "Entire_TME.Rds" data file. Protein-coding genes were retained, ribosomal/mitochondrial genes (starting with "RPS", "RPL" and "MT-") and genes with maximum expression <=4 were removed. In addition, cells from frozen samples (n=3 samples) were removed. The data were normalized using SCTransform[14] and initial zero counts were restored. PCA was performed and Harmony was used to adjust for 3‘ vs 5‘ library chemistry (theta = 6, lambda = 0.1)[11]. Clustering was performed using the Seurat (Seurat 4.3.0) functions FindNeighbors and FindClusters on the top 30 Harmony dimensions (k.param = 15, Louvain algorithm, resolution = 0.2)[15]. UMAP was used to visualize the data and cluster identities were manually assigned using key marker gene expression.

External bulk RNA sequencing data

External bulk RNA sequencing data have been processed as described previously (PMID: 31942071). Briefly, data from Riaz et al.[16] were downloaded as "CountData.BMS038.txt" data file, protein-coding genes were retained, normalized for transcript length, transformed to transcript per million, quantile-normalized and log-transformed. Patients with matched pre/on treatment samples were further analyzed. Data from Gide et al.[17] were obtained from the European Nucleotide Archive (PRJEB23709), and were processed using HISAT and Stringtie[18]

to obtain FPKM values. The data were reduced to protein-coding genes, quantile-normalized and log-transformed. The signature score was calculated as the mean expression of the signature genes. The PD1-treatment RNA-seq data from Gide et al.[17] were downloaded as fastq files from the European Nucleotide Archive (PRJEB23709) and fragments per kilobase of transcript per million mapped reads (FPKM) values were retreived using HISAT and Stringtie. The data were reduced to protein-coding genes, samples were quantile-normalized and the data were log-transformed as log2(data + 1). Previously published PD1 inhibitor-treatment RNA-seq data28 were downloaded as count data ('CountData.BMS038.txt') with annotations from https://github.com/riazn/bms038_analysis/tree/master/data. The data were reduced to protein-coding genes and normalized for transcript lengths using exon annotations from the R package TxDb.Hsapiens.UCSC.hg19.knownGene, subsequently transformed to transcripts per million (TPM) and quantile-normalized. The data were log-transformed as log2(data + 2) − 1.

References

1. Helkkula T, Christensen G, Ingvar C, et al. BioMEL: a translational research biobank of melanocytic lesions and melanoma. *BMJ Open* 2024;14(2):e069694. doi: 10.1136/bmjopen-2022-069694 [published Online First: 20240202]
2. Tsai S, McOlash L, Palen K, et al. Development of primary human pancreatic cancer organoids, matched stromal and immune cells and 3D tumor microenvironment models. *BMC Cancer* 2018;18(1):335. doi: 10.1186/s12885-018-4238-4 [published Online First: 20180327]
3. Cattaneo CM, Dijkstra KK, Fanchi LF, et al. Tumor organoid-T-cell coculture systems. *Nat Protoc* 2020;15(1):15-39. doi: 10.1038/s41596-019-0232-9 [published Online First: 20191218]
4. Vilgelm AE, Bergdorf K, Wolf M, et al. Fine-Needle Aspiration-Based Patient-Derived Cancer Organoids. *iScience* 2020;23(8):101408. doi: 10.1016/j.isci.2020.101408 [published Online First: 20200724]
5. Neal JT, Li X, Zhu J, et al. Organoid Modeling of the Tumor Immune Microenvironment. *Cell* 2018;175(7):1972-88 e16. doi: 10.1016/j.cell.2018.11.021 [published Online First: 2018/12/15]
6. Lauss M, Visne I, Kriegner A, et al. Monitoring of technical variation in quantitative high-throughput datasets. *Cancer Inform* 2013;12:193-201. doi: 10.4137/CIN.S12862
7. Lai Z, Markovets A, Ahdesmaki M, et al. VarDict: a novel and versatile variant caller for next-generation sequencing in cancer research. *Nucleic Acids Res* 2016;44(11):e108. doi: 10.1093/nar/gkw227 [published Online First: 20160407]
8. Wang K, Li M, Hakonarson H. ANNOVAR: functional annotation of genetic variants from high-throughput sequencing data. *Nucleic Acids Res* 2010;38(16):e164. doi: 10.1093/nar/gkq603 [published Online First: 2010/07/06]
9. Bolotin DA, Poslavsky S, Mitrophanov I, et al. MiXCR: software for comprehensive adaptive immunity profiling. *Nat Methods* 2015;12(5):380-1. doi: 10.1038/nmeth.3364

10. Shugay M, Bagaev DV, Turchaninova MA, et al. VDJtools: Unifying Post-analysis of T Cell Receptor Repertoires. *PLoS Comput Biol* 2015;11(11):e1004503. doi: 10.1371/journal.pcbi.1004503 [published Online First: 20151125]
11. Korsunsky I, Millard N, Fan J, et al. Fast, sensitive and accurate integration of single-cell data with Harmony. *Nat Methods* 2019;16(12):1289-96. doi: 10.1038/s41592-019-0619-0 [published Online First: 20191118]
12. Elewaut A, Estivill G, Bayerl F, et al. Cancer cells impair monocyte-mediated T cell stimulation to evade immunity. *Nature* 2025;637(8046):716-25. doi: 10.1038/s41586-024-08257-4 [published Online First: 20241127]
13. Pozniak J, Pedri D, Landeloos E, et al. A TCF4-dependent gene regulatory network confers resistance to immunotherapy in melanoma. *Cell* 2024;187(1):166-83 e25. doi: 10.1016/j.cell.2023.11.037
14. Hafemeister C, Satija R. Normalization and variance stabilization of single-cell RNA-seq data using regularized negative binomial regression. *Genome Biol* 2019;20(1):296. doi: 10.1186/s13059-019-1874-1 [published Online First: 20191223]
15. Butler A, Hoffman P, Smibert P, et al. Integrating single-cell transcriptomic data across different conditions, technologies, and species. *Nat Biotechnol* 2018;36(5):411-20. doi: 10.1038/nbt.4096 [published Online First: 20180402]
16. Riaz N, Havel JJ, Makarov V, et al. Tumor and Microenvironment Evolution during Immunotherapy with Nivolumab. *Cell* 2017;171(4):934-49 e15. doi: 10.1016/j.cell.2017.09.028
17. Gide TN, Quek C, Menzies AM, et al. Distinct Immune Cell Populations Define Response to Anti-PD-1 Monotherapy and Anti-PD-1/Anti-CTLA-4 Combined Therapy. *Cancer Cell* 2019;35(2):238-55 e6. doi: 10.1016/j.ccell.2019.01.003 [published Online First: 2019/02/13]
18. Pertea M, Kim D, Pertea GM, et al. Transcript-level expression analysis of RNA-seq experiments with HISAT, StringTie and Ballgown. *Nat Protoc* 2016;11(9):1650-67. doi: 10.1038/nprot.2016.095 [published Online First: 2016/08/26]
19. Hu M, Coleman S, Judson-Torres RL, et al. The classification of melanocytic gene signatures. *Pigment Cell Melanoma Res* 2024;37(6):854-63. doi: 10.1111/pcmr.13189 [published Online First: 20240728]

Supplementary Figures

Supplementary Figure 1

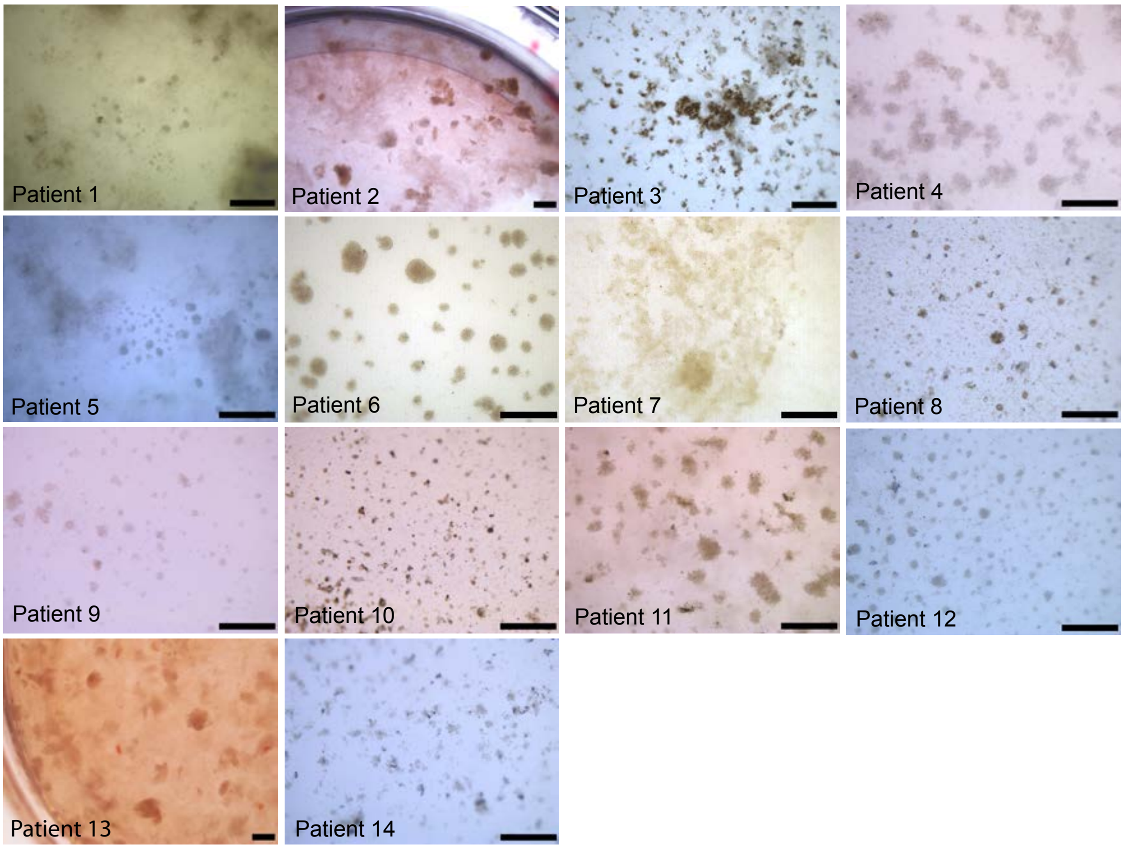

Supplementary Figure 1. Light microscopic images of organotypic cultures from metastatic melanoma specimens.

Supplementary Figure 2

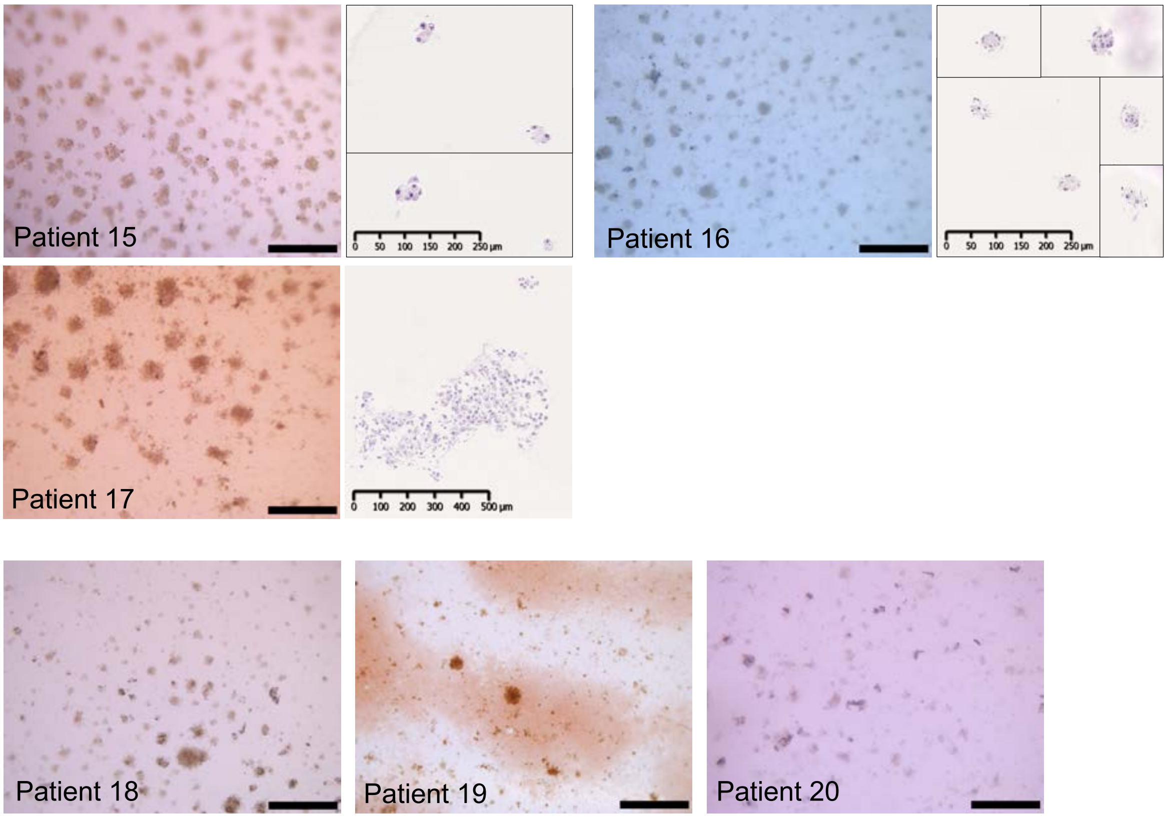

Supplementary Figure 2. Light microscopic images of organotypic cultures from metastatic melanoma specimens not included in the RNA sequencing part of the study.

Supplementary Figure 3

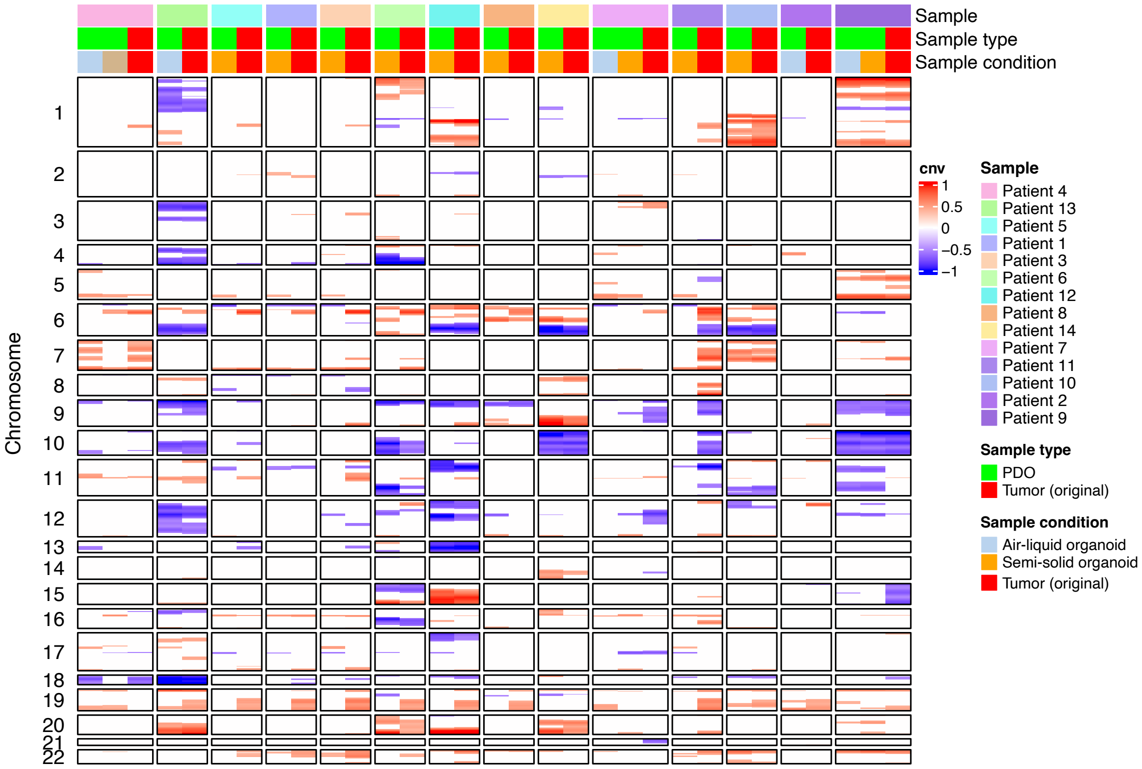

Supplementary Figure 3. Genome-wide DNA copy number variation analysis of matched metastatic lesion and organotypic cultures from 14 melanoma patients. cnv – copy number variation.

Supplementary Figure 4

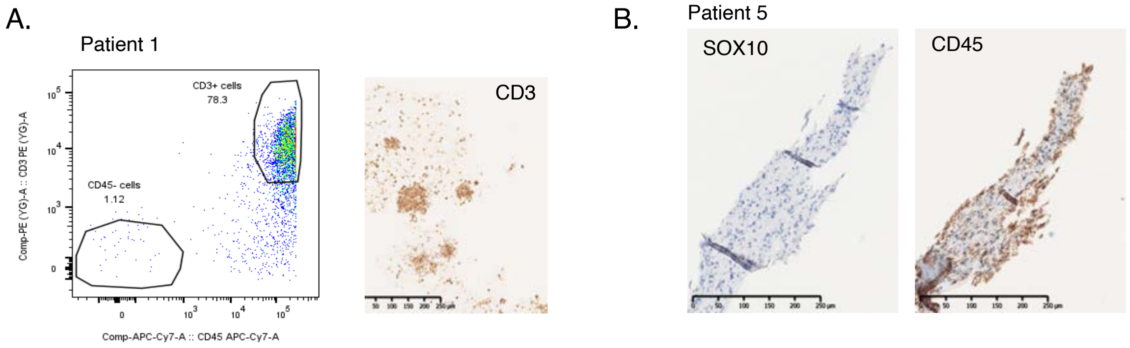

Supplementary Figure 4. Conformation of lack of melanoma cells in organotypic cultures with no copy number variations detected. A) Flow cytometry analysis using CD3 and CD45 analysis shows predominantly CD45+ cells in culture which is confirmed by CD3 immunostaining. B) SOX10 and CD45 immunostaining of organotypic culture from patient 5 shows lack of melanoma cells.

Supplementary Figure 5

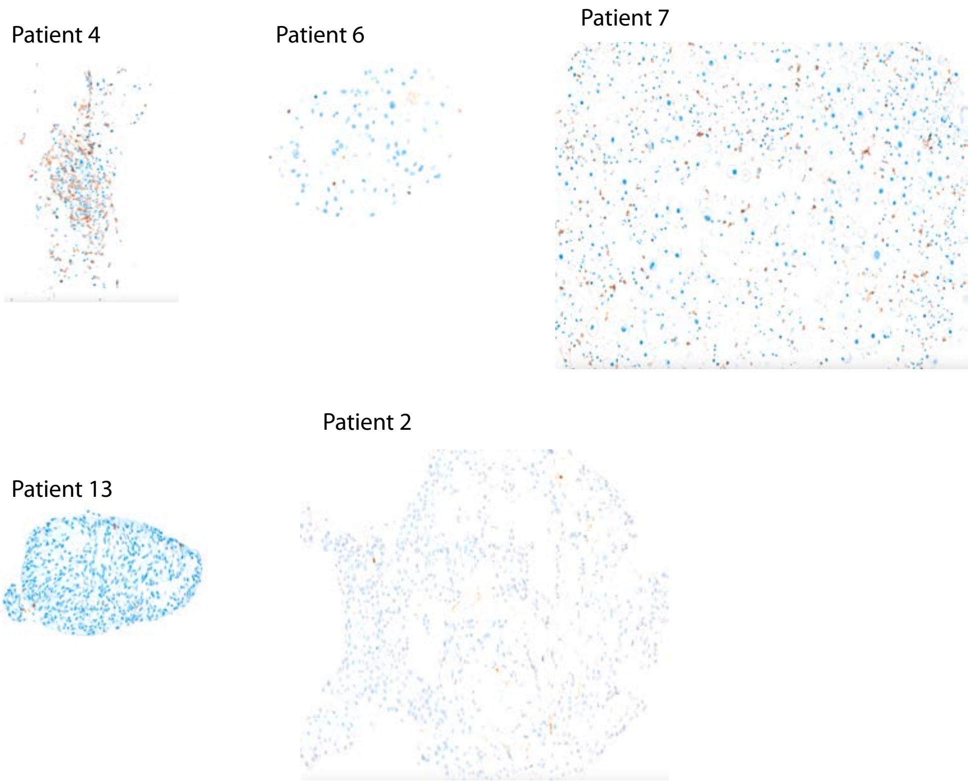

Supplementary Figure 5. Representative examples of organotypic cultures stained with CD8. CD8+ cells are stained in brown color.

Supplementary Figure 6

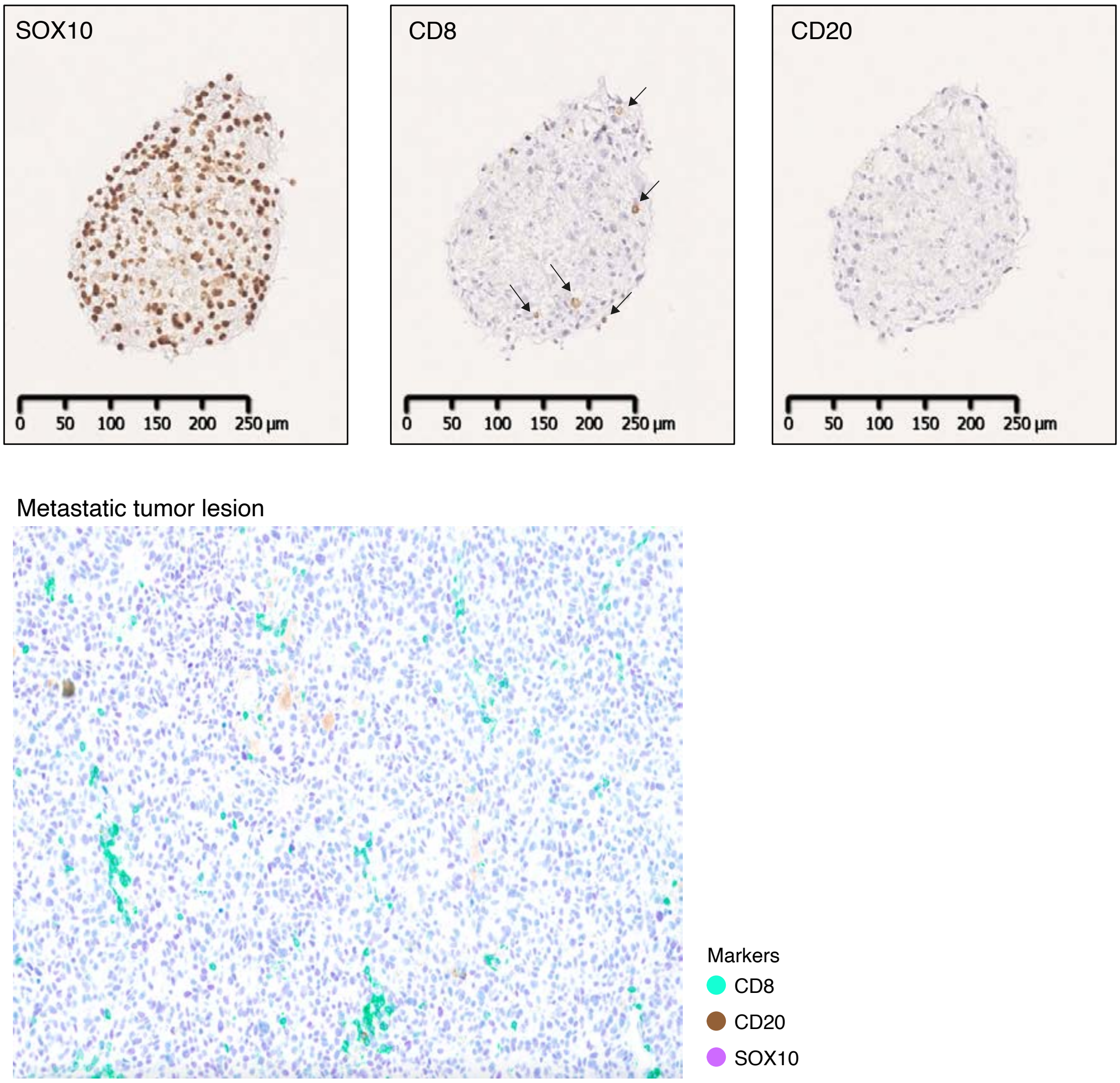

Supplementary Figure 6. Immunostaining of organotypic culture and matched metastatic lesion from patient 6. Upper panels show immunostaining of the organoids with limited infiltration of CD8+ T cells (arrows) and no CD20+ B cells. Lower panel show a triplestaining of CD8, CD20 and SOX10 in the metastatic tissue from patient 6 and from which tissue the organoids were generated from. This shows relatively low abundance of CD8+ T cells (turquoise).

Supplementary Figure 7

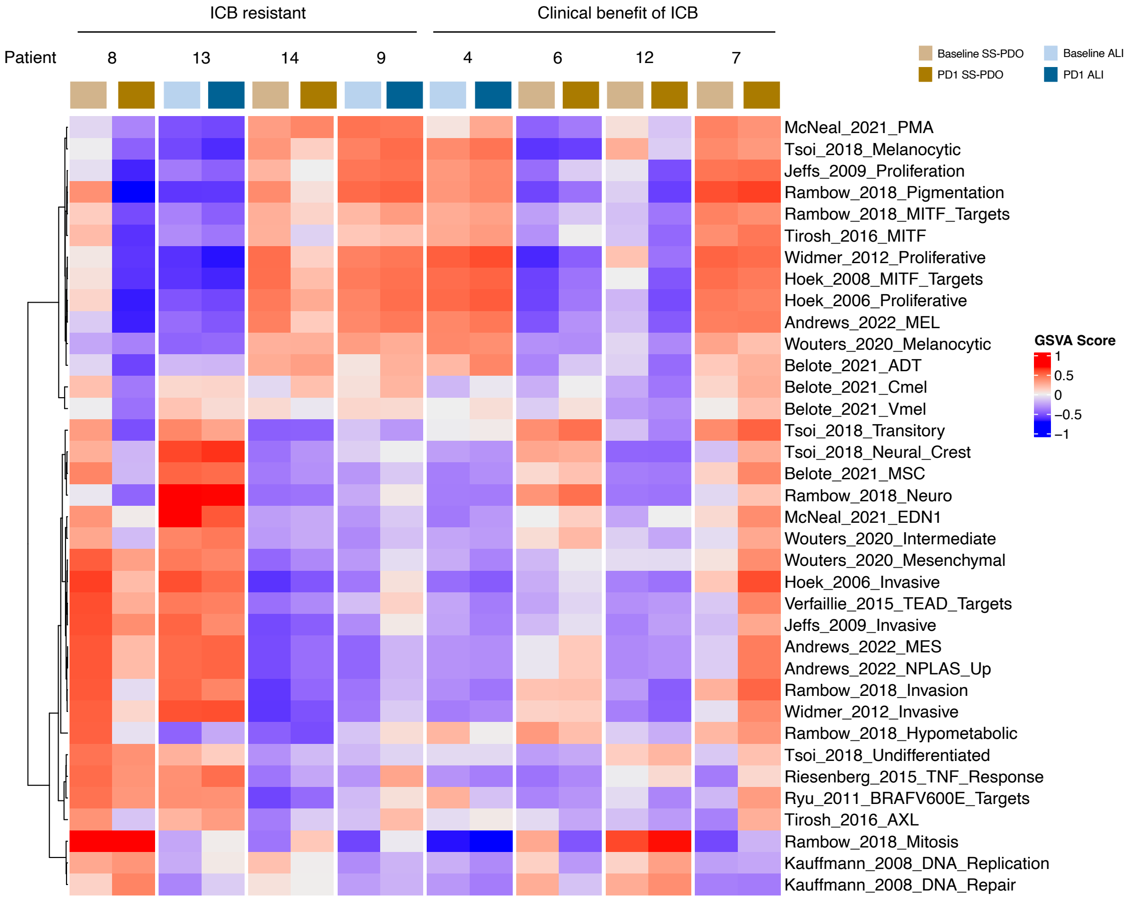

Supplementary Figure 7. Melanocytic gene expression signatures as described by Hu et al.[19]. Heatmap of gene expression scores for each signature of baseline organoids and T cell stimulated organoids from immune checkpoint blockade (ICB) resistant patients (n=4) and baseline and T cell stimulated organoids from ICB responders (n=4).

Supplementary Figure 8

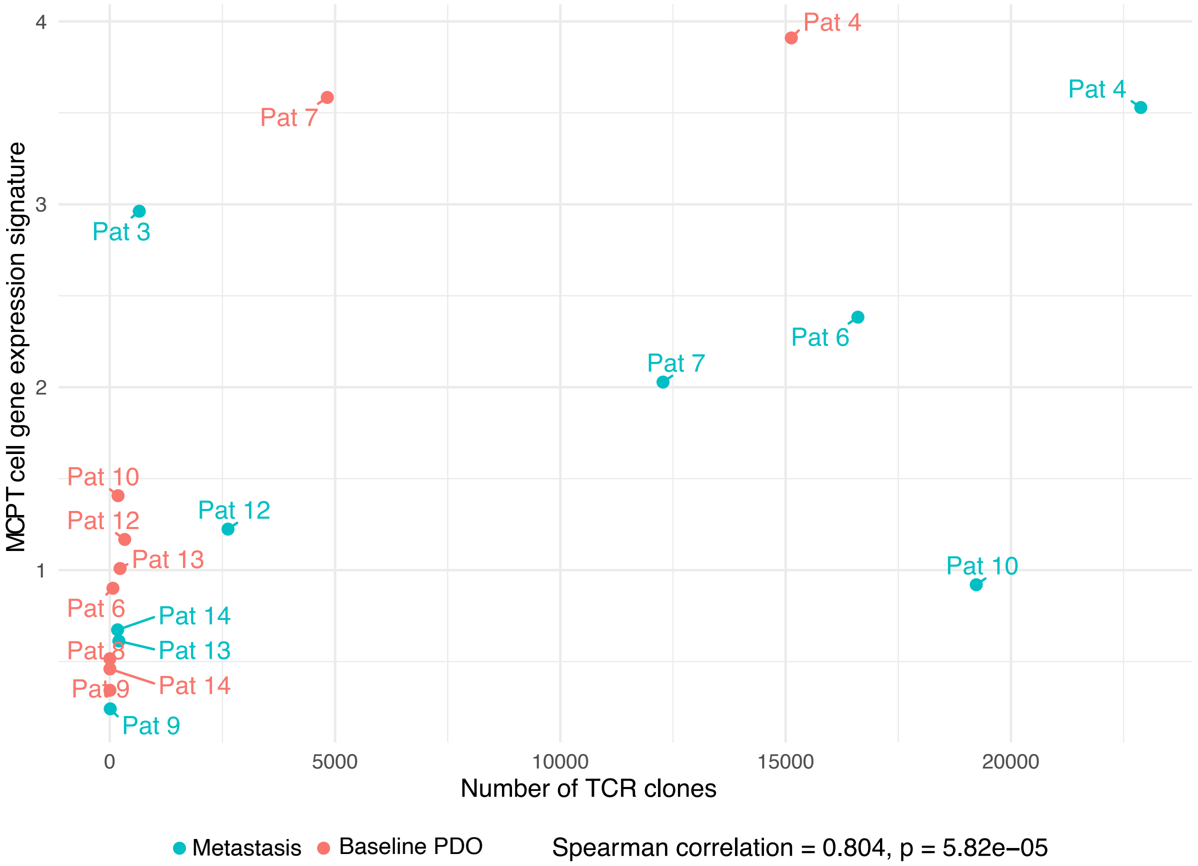

Supplementary Figure 8. Correlation between abundance of T cell receptor clones and T cell gene expression score. Correlation plot of the number of T cell clones (Efron thisted) and MCP T cell gene expression score. Metastatic tumor lesions (n=9) and baseline organoids (n=9) were included in the analysis.

Supplementary Figure 9

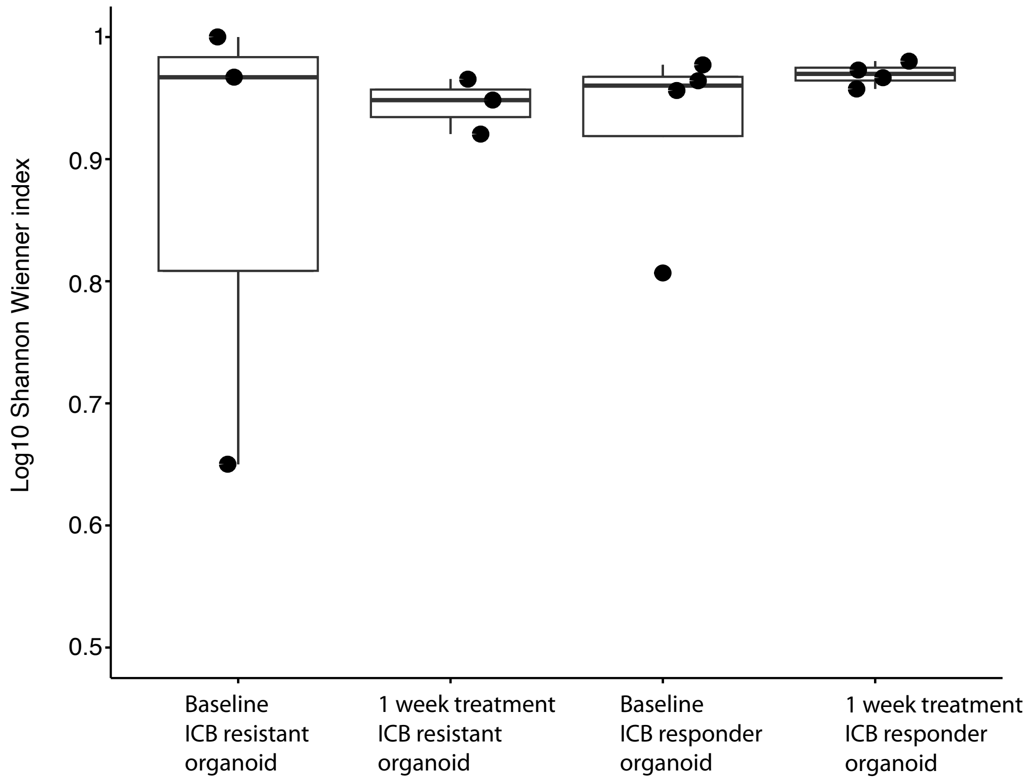

Supplementary Figure 9. T cell receptor diversity measured by Shannon-Wiener index. Boxplot of log10-Shannon-Wiener index of baseline organoids from immune checkpoint blockade (ICB) resistant (n=3), T cell stimulated (CD3, CD28 agonists and PD1 antibody) ICB resistant organoids (n=3), baseline organoids from ICB responders (n=4) and T cell stimulated ICB responder organoids (n=4).

Supplementary Figure 10

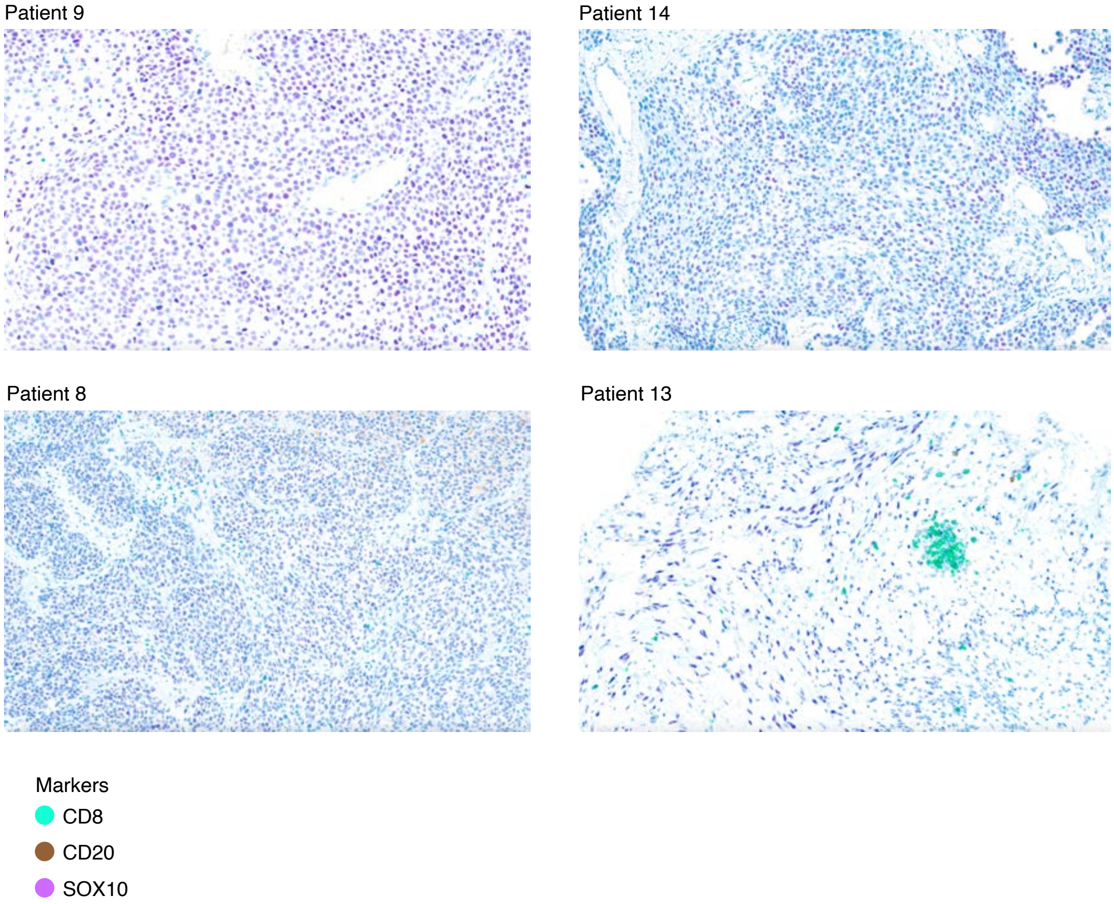

Supplementary Figure 10. Immunostainings of metastatic lesions removed after patient relapsed on immune checkpoint blockade.
